# Association of the Healthy, Hunger-Free Kids Act of 2010 With Body Mass Trajectories of Children in Low-Income Families

**DOI:** 10.1001/jamanetworkopen.2022.10480

**Published:** 2022-05-05

**Authors:** Andrea S. Richardson, Margaret M. Weden, Irineo Cabreros, Ashlesha Datar

**Affiliations:** 1 Department of Behavioral and Policy Sciences, RAND Corporation, Pittsburgh, Pennsylvania; 2 Department of Economics, Sociology, and Statistics, RAND Corporation, Santa Monica, California; 3 Department of Economics, Sociology, and Statistics, RAND Corporation, Boston, Massachusetts; 4 Center for Economic and Social Research, University of Southern California, Los Angeles

## Abstract

**Importance:**

Implemented in 2012, the Healthy, Hunger-Free Kids Act of 2010 (HHFKA) increased nutritional requirements of the National School Lunch Program (NSLP) to reverse the potential role of the NSLP in childhood obesity.

**Objective:**

To evaluate whether associations between the free or reduced-price NSLP and body mass growth differed after implementation of the HHFKA.

**Design, Setting, and Participants:**

This cohort study used data from 2 nationally representative cohorts of US kindergarteners sampled in 1998 to 1999 and 2010 to 2011 and followed up for 6 years, through grade 5, in the Early Childhood Longitudinal Study Kindergarten Class of 1998-1999 (ECLS-K:1999, in 2003-2004) and Kindergarten Class of 2010-2011 (ECLS-K:2011, in 2015-2016). In total, 5958 children were selected for analysis from low-income families eligible for the free or reduced-price NSLP (household income <185% of the federal poverty level) who attended public schools and had no missing data on free or reduced-price NSLP participation or on body mass index (BMI; calculated as weight in kilograms divided by height in meters squared) at kindergarten or grades 1 and 5. Data were analyzed from January 1 to September 7, 2021.

**Exposures:**

Cross-cohort comparison of before vs after implementation of the HHFKA for free or reduced-price NSLP participation at kindergarten and grades 1 and 5.

**Main Outcomes and Measures:**

Body mass index difference (BMID) from obesity threshold was the difference in BMI units from the age- and sex-specific obesity thresholds (95th percentile) and is sensitive to change at high BMI. Multigroup models by cohort included weights to balance the distribution of the 2 cohorts across a wide range of covariates. A Wald test was used to assess whether associations differed between the cohorts.

**Results:**

In the final analysis, 3388 children in ECLS-K:1999 (1696 girls [50.1%]; mean [SD] age at baseline, 74.6 [4.3] months) and 2570 children in ECLS-K:2011 (1348 males [52.5%]; mean [SD] age at baseline, 73.6 [4.2] months) were included. The best fitting model for BMID change by free or reduced-price NSLP participation across the cohorts included fixed and time-varying associations. Before HHFKA implementation, grade 5 free or reduced-price NSLP participants had higher BMID, adjusted for their prior BMID trajectory, than nonparticipants (β = 0.54; 95% CI, 0.27-0.81). After HHFKA implementation, this association was attenuated (β = −0.07; 95% CI, −0.58 to 0.45), and grade 5 associations were different across cohorts (χ^2^_1_ = 4.29, *P* = .04).

**Conclusions and Relevance:**

In this cohort study using cross-cohort comparisons, children from low-income families who participated in the free or reduced-price NSLP had a higher likelihood of progression to high BMI that was no longer observed after HHFKA implementation. This finding suggests that the HHFKA may have attenuated the previous association of the NSLP with child obesity disparities.

## Key Points

**Question:**

Has the Healthy, Hunger-Free Kids Act of 2010 (HHFKA) improved body mass trajectories of children in low-income families?

**Findings:**

In this cohort study evaluating 3388 children before HHFKA implementation and 2570 children after HHFKA implementation, kindergarteners in low-income families who did not participate in the free or reduced-price National School Lunch Program before the HHFKA had steeper decreases in body mass index (BMI) difference from obesity threshold between grades 1 and 5 than their participating peers. After HHFKA implementation, this obesogenic association between the National School Lunch Program and BMI difference change was no longer observed.

**Meaning:**

These findings suggest that implementation of the HHFKA may have helped curb obesogenic increase in BMI among low-income children.

## Teaser

This cohort study uses data from 2 nationally representative cohorts of US kindergarteners followed up through grade 5 to assess whether body mass growth differed after implementation of the Healthy, Hunger-Free Kids Act of 2010 among children in low-income families.

## Introduction

Obesity remains high among children,^1^ and severe obesity is climbing,^2^ especially in low-income populations.^3,4^ Although school meals are a critical source of nutrients for low-income children,^5^ growing evidence suggests that prior to 2010, school meals may have contributed to childhood obesity.^6,7,8,9,10^

Congress passed the Healthy, Hunger-Free Kids Act of 2010 (HHFKA)^11^ to reduce childhood obesity by increasing the nutritional requirements in school meals. Since its implementation in 2012, growing evidence suggests that the HHFKA improved children’s dietary quality^12,13,14,15,16,17,18^; however, to our knowledge, only 2 observational studies have estimated associations with child growth, and those had mixed findings.^19,20^ An interrupted time series of National Survey of Children’s Health (2003-2018) observed decreased obesity trends after HHFKA implementation among impoverished children.^20^ In a multivariate regression analysis assessing data before and after HHFKA implementation in the Early Childhood Longitudinal Study (ECLS) of children in grades 1 and 3, the HHFKA was associated with slower body mass index (BMI; calculated as weight in kilograms divided by height in meters squared) *z* score growth in grade 3 but only for boys.^19^ However, those studies’ findings may not be robust to problems of selection bias in observational data. Several studies before HHFKA implementation have shown that ignoring selection into the National School Lunch Program (NSLP) biases associations downward between the NSLP and child weight.^7,8,9,21^ The 2 studies that examined changes in child growth before and after HHFKA implementation used BMI *z* score^19^ and obesity^20^ outcomes, which raises concerns about their findings given BMI *z* score limitations for capturing extreme values^22,23,24,25,26^ and severe obesity increases,^2,27,28^ especially in low-income populations.^29^ We evaluated 2 national cohorts of children followed throughout elementary school to examine whether associations between free or reduced-price NSLP participation and body mass growth of children from low-income families changed following HHFKA implementation. We followed the Centers for Disease Control and Prevention guidelines^30^; thus, our analysis is sensitive to extreme BMI values that are due to severe obesity increases.^2,27,28,29^ We account for body mass tracking over time,^31,32,33,34^ and we model differences within child and between children to reduce selection bias.

## Methods

### Participants

We used data from cohorts of nationally representative samples of US kindergarteners in ECLS. The ECLS Kindergarten Class of 1998-1999 (ECLS-K:1999) recruited 21 409 kindergartens in fall of 1998 from 1000 public and private schools, and the ECLS-K:2011 recruited 18 174 kindergarteners in fall of 2010 from 970 public and private schools across the US. Both cohort studies followed up children through grade 5. The ECLS obtained parent consent before data collection. Our analyses are based on data collected in kindergarten and grades 1 and 5, when both cohort studies surveyed parents about their child’s NSLP participation. The present study followed the Strengthening the Reporting of Observational Studies in Epidemiology (STROBE) reporting guideline for cohort studies. The RAND Human Subjects Protection Committee approved this study and waived the need for informed consent per the Common Rule.

**Figure 1.**
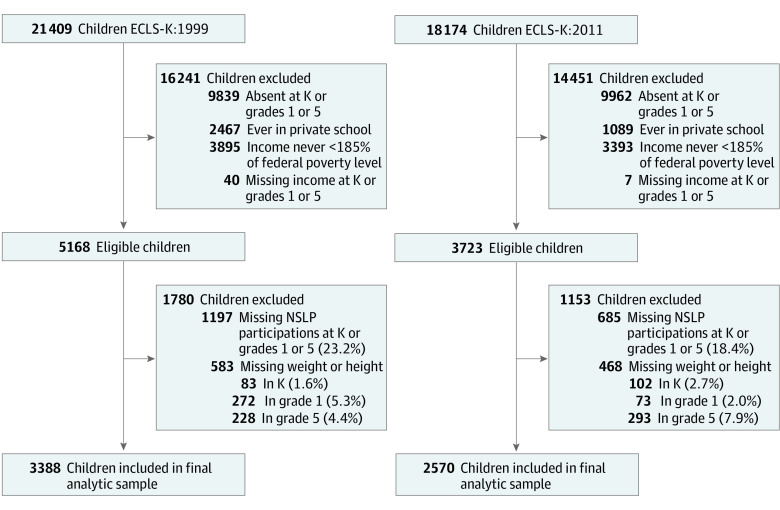
Flow of Analytic Samples in Early Childhood Longitudinal Study Kindergarten Class of 1998-1999 (ECLS-K:1999) and Kindergarten Class of 2010-2011 (ECLS-K:2011) Cohorts NSLP represents free or reduced-price National School Lunch Program.

Of 21 409 children (ECLS-K:1999) and 18 174 children (ECLS-K:2011) recruited for the studies, children were ineligible for the present study if they did not participate in any of the waves (kindergarten and grades 1 and 5) (9839 children in ECLS-K:1999 and 9962 children in ECLS-K:2011), if they ever attended private school (2467 in ECLS-K:1999 and 1089 children in ECLS-K:2011), if they never qualified for the free or reduced-price NSLP (ie, income never <185% of the federal poverty level at kindergarten, and grades 1 and 5)^35^ (3895 children in ECLS-K:1999 and 3393 children in ECLS-K:2011), or if they had missing household income at kindergarten or grades 1 and 5 (40 children in ECLS-K:1999 and 7 children in ECLS-K:2011) (Figure 1). Of 5168 eligible children in ELCSK:1999 and 3723 eligible children in ECLS-K:2011, we excluded those with missing NSLP data (1197 children [23.2%] in ECLS-K:1999 and 685 children [18.4%] in ECLS-K:2011) or BMI score (83 children in kindergarten [1.6%], 272 children in grade 1 [5.3%], and 228 children in grade 5 [4.4%] in ECLS-K:1999; and 102 children in kindergarten [2.7%], 73 children in grade 1 [2.0%], and 293 children in grade 5 [7.9%] in ECLS-K:2011). The study design and children’s changing schools reduced grade 1 and 5 samples, explaining most nonresponses.^36,37^ Compared with the analytic sample, eligible children excluded for missing BMI data differed significantly from children not excluded with respect to race and ethnicity, urbanicity, family dinners (ECLS-K:1999 only), and mother's educational level (ECLS-K:1999 only), but there were no significant differences in free or reduced-price NSLP participation, household income, child's birth weight, or mother's work status (eTable 1 in the Supplement). However, differences may be spurious owing to multiple testing. Compared with the analytic sample, BMI at kindergarten was similar for excluded children (500 children in ECLS-K:1999: difference in means, 0.1 (95% CI, −0.1 to 0.4); 366 children in ECLS-K:2011: difference in means, −0.1 (95% CI, −0.4 to 0.2).

### Outcomes

During the spring of each wave, trained assessors measured children’s height (to the nearest quarter inch using a ShorrBoard; Weigh and Measure LLC) and weight (to the nearest half-pound using a Seca digital bathroom scale). The BMI percentiles and *z* scores have not matched empirical data for children with high BMI.^22,23,24,25,26^ Consequently, high BMIs are conflated to narrow distributions of BMI *z* scores and percentiles. Thus, BMI measures relative to the 95th percentile are better than BMI *z* scores or percentiles at identifying children with severe obesity.^25^ We used the SAS macro from the Centers for Disease Control and Prevention to calculate the difference between the child’s BMI and the 95th percentile BMI value (BMID) for sex and age.^30^ We describe BMI *z* scores and weight status using BMI percent of the 95th percentile (BMIP) for underweight (<5.3 BMIP), normal weight (5.3 to <89 BMIP), overweight (89 to <100 BMIP), obesity (100 to <120 BMIP), and severe obesity (≥120 BMIP).

### Exposures

During fall of kindergarten and grades 1 and 5, the ECLS-K:1999 parent responded to 2 questions: “Does (CHILD) usually receive a complete lunch offered at school? By complete school lunch, I mean a complete meal such as a salad, soup, a sandwich, or a hot meal that is offered each day at a fixed price, not just milk, snacks, ice cream, or a lunch (he/she) brought from home”; and “Are these lunches free or reduced price?” Although ECLS-K:2011 parent surveys at kindergarten and grade 1 included the same questions, at grade 5 the parents were asked “Does (CHILD) receive complete school lunches for free or reduced price at school?” For consistency, we used free or reduced-price lunch participation to classify NSLP participation. The NSLP lunches were the same regardless of the price, yet school lunches may provide low-income children a greater proportion of their daily nutritional intake because low-income households may have lower nutritional quality than school lunches.^38^ We hypothesize that the association of the HHFKA with children’s dietary quality may be greater for children in low-income families than their more advantaged peers.

### Covariates

Unless specified below, ECLS-K:1999 and ECLS-K:2011 used the same survey instruments.^39,40^ During the fall kindergarten wave, guardians reported time-invariant characteristics: child’s sex, race and ethnicity (Black non-Hispanic, Hispanic, White non-Hispanic, and other [American Indian or Alaska Native, Asian, Native Hawaiian or Pacific Islander, or more than 1 race and ethnicity]), birthweight, and mother’s highest level of education. Reported time-varying characteristics included child’s age, parent’s employment status in the last week (eg, “work at a job for pay”), whether “in a typical week” the family “eats the evening meal together,” number of hours a day the child “usually watch[es] TV or videos on school days” (not asked in ECLS-K:2011 for grade 5), and last year’s household income. We chose these covariates because they could be associated with unobserved factors that influence free or reduced-price NSLP participation and family norms that may increase obesity risk, such as watching television and eating habits.^9,41,42,43^ School urbanicity was linked to the cohort by the National Center for Education Statistics prior to releasing the data set.^39^

### Statistical Analysis

Descriptive statistics were obtained with Stata, version 15.1 (StataCorp LLC). The HHFKA was first implemented in grade 2 of ECLS-K:2011, which enabled us to examine how the HHFKA may have changed associations between free or reduced-price NSLP and BMID trajectories. We made 3 comparisons to reduce bias: (1) within-child change in BMID; (2) differences in free or reduced-price NSLP associations with BMID change (grades 1 vs 5); and (3) differences between cohorts. The historical cohort enabled us to observe how changes in BMID over time between free or reduced-price NSLP and non-NSLP participants evolved before the HHFKA.

We used a general panel approach with structural equation modeling^44^ and tested which model best fit the data. We tested a fixed-effects model, a random-effects model, and a model with time varying and time-invariant coefficients, with or without lagged outcomes. The best fitting model for BMID change by free or reduced-price NSLP participation across the cohorts included fixed and time-varying associations (eTable 2 and eTable 3 in the Supplement). This allowed for associations between BMID and time-varying characteristics to change as children aged, adjusted for BMID in the previous waves and adjusted for time-invariant child characteristics (eg, birth weight). Because children’s heights and weights were measured in the spring, we posited that this timing provided time for participation to have a lagged effect on body mass. Furthermore, because BMID at time *t* is a function of BMID at time *t*−1, it is also a function of the association between lagged BMI at time *t*−1 and NSLP at time *t*−1. More details regarding the chosen model, commands, output, and mathematical specifications are given in eMethods in the Supplement.

We use entropy balancing^45^ to ensure that HHFKA-exposed and HHFKA-unexposed cohorts were comparable (eMethods in the Supplement). Weighted cohorts were similar across covariates, whereas unweighted cohorts had discrepancies (eTables 4 and 5 in the Supplement). We used Mplus, version 7.11,^44^ and missing covariate data were handled with multiple imputation using the mice package in R.^46^ Statistical significance was defined by a 2-sided *P* value <.05. We plotted estimated mean BMIDs by free or reduced-price NSLP participation and report standard errors.

We examined whether associations varied by sex but did not assess associations by race and ethnicity because of low statistical power. We conducted sensitivity analyses, including imputation of grade 5 full-price NSLP participation in ECLS-K:2011 (eMethods and eTable 6 in the Supplement). All data were analyzed from January 1 to September 7, 2021.

## Results

In total, 3388 children in ECLS-K:1999 (1696 girls [50.1%] and 1692 boys [49.9%]; mean [SD] age at baseline, 74.6 [4.3] months) and 2570 children in ECLS-K:2011 (1222 girls [47.5%] and 1348 boys [52.5%]; mean [SD] age at baseline, 73.6 [4.2] months) were included in the analysis. The data given in the Table reflect severe obesity among US kindergarteners from low-income families, providing evidence that BMID is an appropriate measure to study body mass growth.^30^ The ECLS-K:1999 kindergarteners’ mean BMI was 2 BMI units below their obesity threshold. Between ECLS-K:1999 and ECLS-K:2011, the mean BMID of kindergarteners from low-income families moved closer to their obesity threshold. Free or reduced-price NSLP participation increased in ECLS-K:1999 from 1896 (56.0%) to 2309 (68.2%) and from 1767 (68.8%) to 1957 (76.7%) in ECLS-K:2011. The median household income decreased from $22 500 (IQR, $12 500-$32 500) in 1999 to $20 522 (IQR, $13 060-$27 985) in 2011, after adjusting for inflation. However, mother’s highest achieved educational level at kindergarten increased between the cohorts, with an approximately 10 percentage-point increase in at least some college attainment. The more recent cohort (ECLS-K:2011) included more Hispanic children (1026 [39.9%] vs 955 [28.2%]) and fewer non-Hispanic Black (605 [17.9%] vs 280 [10.9%]) or non-Hispanic White (1418 [41.9%] vs 979 [38.1%]) children than in ECLS-K:1999.

**Table.** Baseline Characteristics of ECLS Cohorts by Free or Reduced-Price National School Lunch Program Participation

Characteristic	No. (%)					
	ECLS-K:1999			ECLS-K:2011		
	All (n = 3388)	NSLP		All (n = 2570)	NSLP	
		Not free or reduced price^a^	Free or reduced price^b^		Not free or reduced price^c^	Free or reduced price^d^
Age in kindergarten, mean (SD), mo	74.6 (4.3)	74.9 (4.3)	74.4 (4.2)	73.6 (4.5)	73.7 (4.5)	73.6 (4.6)
Race and ethnicity						
Black non-Hispanic	605 (17.9)	129 (8.7)	476 (25.1)	280 (10.9)	29 (3.6)	251 (14.2)
Hispanic	955 (28.2)	277 (18.6)	678 (35.8)	1026 (39.9)	171 (21.3)	855 (48.4)
White non-Hispanic	1418 (41.9)	925 (62.0)	493 (26.0)	979 (38.1)	480 (59.8)	499 (28.2)
Other^e^	409 (12.1)	161 (10.8)	248 (13.1)	285 (11.1)	123 (15.3)	162 (9.2)
Missing	1 (0.03)	0	1 (0.1)	0	0	0
Sex						
Female	1696 (50.1)	731 (49.0)	965 (50.9)	1222 (47.6)	375 (46.7)	847 (47.9)
Male	1692 (49.1)	761 (51.0)	931 (49.1)	1348 (52.5)	428 (53.3)	920 (52.1)
BMID, mean (SD)^f^						
Kindergarten	−2.1 (2.6)	−2.3 (2.5)	−2.0 (2.6)	−1.8 (2.6)	−2.1 (2.5)	−1.7 (2.7)
Grade 1	−2.4 (3.2)	−2.7 (2.9)	−2.3 (3.4)	−2.1 (3.2)	−2.6 (2.9)	−1.9 (3.2)
Grade 5	−2.6 (5.2)	−3.1 (5.0)	−2.4 (5.3)	−2.6 (5.2)	−3.7 (5.0)	−2.3 (5.3)
BMI *z* score, mean (SD)						
Kindergarten	0.5 (1.1)	0.4 (1.1)	0.5 (1.2)	0.6 (1.1)	0.5 (1.0)	0.7 (1.1)
Grade 1	0.4 (1.3)	0.4 (1.2)	0.5 (1.4)	0.6 (1.2)	0.4 (1.1)	0.6 (1.2)
Grade 5	0.8 (1.2)	0.7 (1.1)	0.8 (1.2)	0.7 (1.4)	0.5 (1.2)	0.8 (1.5)
Kindergarten weight status						
Underweight	0	0	0	0	0	0
Normal weight	2144 (63.3)	980 (65.7)	1164 (61.4)	1456 (56.7)	502 (62.5)	954 (54.0)
Overweight	778 (23.0)	333 (22.3)	445 (23.5)	672 (26.2)	196 (24.4)	476 (26.9)
Obesity	323 (9.5)	123 (8.2)	200 (10.6)	311 (12.1)	70 (8.7)	241 (13.6)
Severe obesity	143 (4.2)	56 (3.8)	87 (4.6)	131 (5.1)	35 (4.4)	96 (5.4)
Grade 1 weight status						
Underweight	0	0	0	0	0	0
Normal weight	2275 (67.2)	802 (69.9)	1473 (65.3)	1586 (61.7)	414 (67.5)	1172 (59.9)
Overweight	573 (16.9)	197 (17.2)	376 (16.8)	487 (19.0)	112 (18.3)	375 (19.2)
Obesity	339 (10.0)	95 (8.3)	244 (10.9)	345 (13.4)	64 (10.4)	281 (14.4)
Severe obesity	201 (5.9)	54 (4.7)	147 (6.6)	152 (5.9)	23 (3.8)	129 (6.6)
Grade 5 weight status						
Underweight	0	0	0	0	0	0
Normal weight	2000 (59.0)	681 (63.1)	1319 (57.1)	1495 (58.2)	400 (66.8)	1095 (55.6)
Overweight	518 (15.3)	159 (14.7)	359 (15.6)	388 (15.1)	85 (14.2)	303 (15.4)
Obesity	544 (16.1)	153 (14.2)	391 (16.9)	435 (16.9)	84 (14.0)	351 (17.8)
Severe obesity	326 (9.6)	86 (8.0)	240 (10.4)	252 (9.8)	30 (5.0)	222 (11.3)
Birthweight, g						
<2500	299 (8.8)	102 (6.8)	197 (10.4)	182 (7.1)	52 (6.5)	130 (7.4)
2500-3999	2601 (76.8)	1169 (78.4)	1432 (75.5)	1503 (58.5)	504 (62.8)	999 (56.5)
≥4000	355 (10.5)	174 (11.7)	181 (9.6)	193 (7.5)	60 (7.5)	133 (7.5)
Missing	133 (3.9)	47 (3.2)	86 (4.5)	692 (26.9)	187 (23.3)	505 (28.6)
Mother’s educational attainment at kindergarten						
<9th grade	323 (9.5)	65 (4.4)	258 (13.6)	278 (10.8)	40 (5.0)	238 (13.5)
Grade 9-12	503 (14.9)	141 (9.5)	362 (19.1)	344 (13.4)	42 (5.2)	302 (17.1)
High school or GED	1333 (39.3)	588 (39.4)	745 (39.3)	764 (29.7)	197 (24.5)	567 (32.1)
Some college	944 (27.9)	510 (34.2)	434 (22.9)	853 (33.2)	333 (41.5)	520 (29.4)
Bachelor’s degree	160 (4.7)	109 (7.3)	51 (2.7)	240 (9.3)	137 (17.1)	103 (5.8)
≥Graduate school	61 (1.8)	48 (3.2)	13 (0.7)	91 (3.5)	54 (6.7)	37 (2.1)
Missing	64 (1.9)	31 (2.1)	33 (1.7)	0	0	0
Household income, median (IQR), $^g^						
Kindergarten	22 500 (12 500-32 500)	27 500 (17 500-37 500)	17 500 (12 500-27 500)	20 522 (13 060-27 985)	27 985 (20 522-46 642)	16 791 (9328-24 253)
Grade 1	22 500 (12 500-32 500)	32 500 (27 500-45 000)	17 500 (12 500-27 500)	20 522 (13 060-27 985)	33 582 (24 253-46 641)	16 791 (9328-24 253)
Grade 5	27 500 (17 500-37 500)	37 500 (27 500-62 500)	22 500 (12 500-32 500)	24 254 (16 791-33 582)	46 642 (33 582-65 299)	20 522 (13 060-27 985)
Family dinners, mean (SD), No./wk^h^						
Kindergarten	5.8 (1.8)	5.8 (1.8)	5.8 (1.8)	5.8 (1.7)	5.8 (1.7)	5.9 (1.8)
Grade 1	5.9 (1.7)	5.7 (1.7)	5.9 (1.7)	5.7 (1.8)	5.7 (1.7)	5.7 (1.8)
Grade 5	5.6 (1.8)	5.5 (1.8)	5.6 (1.8)	5.6 (1.8)	5.4 (1.7)	5.6 (1.8)
Television, mean (SD), h/d^i^						
Kindergarten	2.1 (1.4)	2.0 (1.3)	2.2 (1.6)	2.3 (1.5)	2.2 (1.5)	2.3 (1.5)
Grade 1	2.3 (1.5)	2.1 (1.3)	2.4 (1.5)	1.8 (1.4)	1.6 (1.2)	1.8 (1.4)
School in urban area^j^						
Kindergarten	1270 (37.5)	446 (29.9)	824 (43.4)	914 (35.6)	189 (23.5)	725 (41.0)
Grade 1	1261 (37.2)	337 (29.4)	924 (41.3)	920 (35.8)	149 (24.3)	771 (39.4)
Grade 5	1202 (35.5)	306 (28.4)	896 (38.8)	809 (31.5)	118 (19.7)	691 (35.1)
Mother employed^k^						
Kindergarten	1258 (37.1)	594 (39.8)	664 (35.0)	644 (25.1)	218 (27.2)	426 (24.1)
Grade 1	1537 (46.8)	555 (48.3)	982 (43.8)	911 (35.5)	260 (42.4)	651 (33.3)
Grade 5	1630 (48.1)	583 (54.0)	1047 (45.3)	1117 (43.5)	330 (55.1)	787 (39.9)

Abbreviations: BMI, body mass index (calculated as weight in kilograms divided by height in meters squared); BMID, difference from obesity threshold (95th percentile); ECLS, Early Childhood Longitudinal Study; ECLS-K:1999, ECLS Kindergarten Class of 1998-1999; ECLS-K:2011, ECLS Kindergarten Class of 2010-2011; NSLP, National School Lunch Program.

^a^ No. (%) in kindergarten, 1492 (44.0%); grade 1, 1148 (33.9%); and grade 5, 1079 (31.8%).

^b^ No. (%) in kindergarten, 1896 (56.0%); grade 1, 2240 (66.1%); and grade 5, 2309 (68.2%).

^c^ No. (%) in kindergarten, 803 (31.2%); grade 1, 613 (23.8%); and grade 5, 599 (23.3%).

^d^ No. (%) in kindergarten, 1767 (68.8%), grade 1, 1957 (76.1%); and grade 5, 1971 (76.7%).

^e^ American Indian or Alaska Native, Asian, Native Hawaiian or Pacific Islander, or more than 1 race and ethnicity.

^f^ BMID is child’s BMI minus the BMI at the age- and sex-specific 95th percentile.

^g^ Household incomes in ECLS-K:2011 were adjusted for inflation from 1999.

^h^ No. missing in ECLS-K:1999 kindergarten, 2; grade 1, 0; and grade 5, 2; and No. missing in ECLS-K:2011 kindergarten, 1; grade 1, 2; and grade 5, 0.

^i^ No. missing in ECLS-K:1999 kindergarten, 36; and grade 1, 0. No. missing in ECLS-K:2011 kindergarten, 0; and grade 1, 0.

^j^ No. missing in ECLS-K:1999 kindergarten, 0; grade 1, 17; and grade 5, 135. No. missing in ECLS-K:2011 kindergarten, 16; grade 1, 40; and grade 5, 84.

^k^ No. missing in ECLS-K:1999 kindergarten, 424; grade 1, 103; and grade 5, 117. No. missing in ECLS-K:2011 kindergarten, 573; grade 1, 0; and grade 5, 0.

At grade 1, there were no associations between BMID and the free or reduced-price NSLP after adjustment for kindergarten BMID and covariates in either ECLS-K:1999 (β = −0.25; 95% CI, −1.17 to 0.68) and ECLS-K:2011 (β = 0.08; 95% CI, −1.04 to 1.20) (eFigure 1 in the Supplement). By grade 5, free or reduced-price NSLP recipients had higher BMID, adjusted for grade 1 BMID, in ECLS-K:1999 (β = 0.54; 95% CI, 0.27-0.81), but there was no association in ECLS-K:2011 (β = −0.07; 95% CI, −0.58 to 0.45). Change in BMID for NSLP recipients between kindergarten and grade 1 was not different between cohorts (χ^2^_1_ = 0.19, *P* = .66). However, the before vs after HHFKA change between cohorts (grades 1 and 5) was different (χ^2^_1_ = 4.29, *P* = .04).

**Figure 2.**
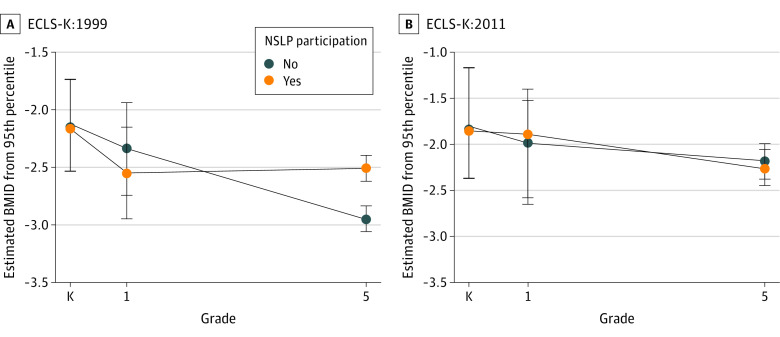
Body Mass Index Difference (BMID) From Obesity by Free or Reduced-Price National School Lunch Program (NSLP) Participation and Early Childhood Longitudinal Study (ECLS) Cohort ECLS-K:1999 indicates ECLS Kindergarten Class of 1998-1999; ECLS-K:2011, ECLS Kindergarten Class of 2010-2011. Error bars represent SEs.

**Figure 3.**
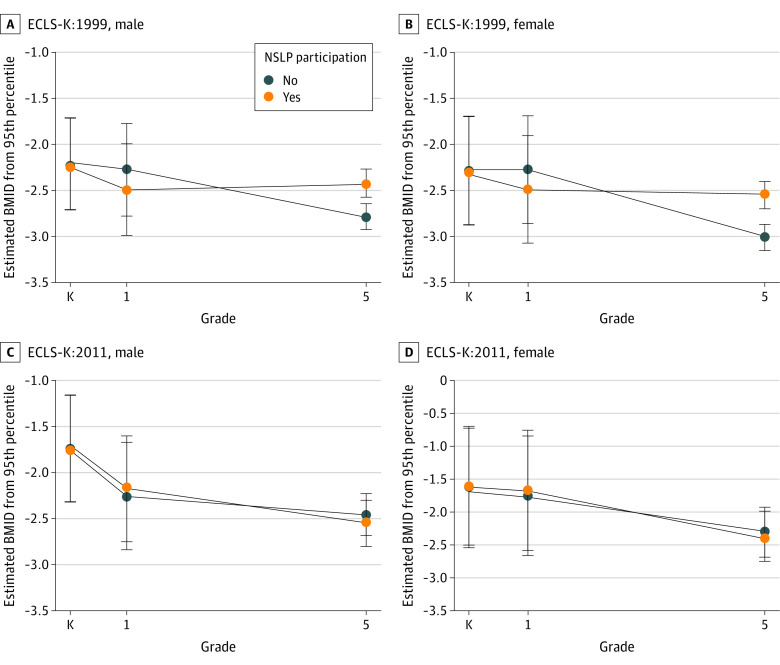
Body Mass Index Difference (BMID) From Obesity by Free or Reduced-Price National School Lunch Program (NSLP) Participation, Early Childhood Longitudinal Study (ECLS) Cohort, and Sex ECLS-K:1999 indicates ECLS Kindergarten Class of 1998-1999; ECLS-K:2011, ECLS Kindergarten Class of 2010-2011. Error bars represent SEs.

**Figure 4.**
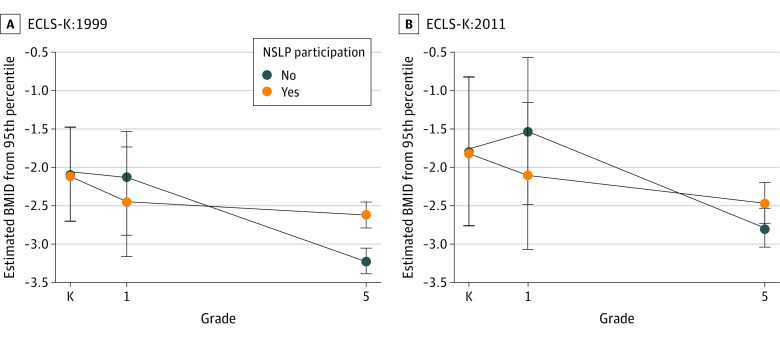
Body Mass Index Difference (BMID) from Obesity by Imputed Full-Price, Free, or Reduced-Price National School Lunch Program (NSLP) Participation and Early Childhood Longitudinal Study (ECLS) Cohort ECLS-K:1999 indicates ECLS Kindergarten Class of 1998-1999; ECLS-K:2011, ECLS Kindergarten Class of 2010-2011. Error bars represent SEs.

Figure 2 presents kindergarten and grades 1 and 5 mean BMID estimates for free or reduced price by NSLP participation and by cohort. Prior to the HHFKA, children participating in the free or reduced-price NSLP maintained their BMID levels from grade 1 through grade 5 compared with nonparticipating children who experienced a decrease in BMID. After the HHFKA, all children had the same declining BMID trajectory. The estimated BMIDs and SEs indicated a negative BMID trajectory in ECLS-K:2011, but BMID change was indistinguishable between the grade 1 and grade 5 years irrespective of the free or reduced-price NSLP. Notably, children in ECLS-K:2011 had higher BMIDs (mean [SE], −1.8 [0.6]) in kindergarten than children in ECLS-K:1999 (mean [SE], −2.2 [0.5]). Furthermore, nonparticipants did not have as steep a BMID decrease as their ECLS-K:1999 counterparts. Associations of before vs after HHFKA BMID change did not differ by sex (χ^2^_1_, 1.84; *P* = .19) (Figure 3). Parents in ECLS-K:1999 reported that 2385 children in kindergarten (70.4%), 3021 children in grade 1 (89.2%), and 3060 children in grade 5 (90.3%) participated in either the full-price, free, or reduced-price NSLP. In ECLS-K:2011, 2043 children in kindergarten (79.5%) and 2257 children in grade 1 (87.8%) participated in the NSLP. After NSLP participation imputation at grade 5 in ECLS-K:2011, we classified 2424 children (94.3%) as participating in the NSLP. The NSLP participation associations were the same as for our main model (Figure 4). For grade 1, there were no associations between BMID and NSLP participation in ECLS-K:1999 (β = −0.21; 95% CI, −1.43 to 1.00) or in ECLS-K:2011 (β = −0.62; 95% CI, −2.87 to 1.63). By grade 5, NSLP recipients in ECLS-K:1999 had higher BMID, adjusted for BMID in grade 1, (β = 0.55; 95% CI, 0.19-0.91), but there was no association in ECLS-K:2011 (β = 0.36; 95% CI, −0.46 to 1.18). The change before vs after the HHFKA from grades 1 to 5 between the cohorts was similar (χ^2^_1_ = 0.17, *P* = .68).

Using BMI *z* scores, the associations were similar with our main model. However, the obesogenic pre-HHFKA grade 5 association between free or reduced-price NSLP and change in BMI *z* score was attenuated (β = 0.08; 95% CI, 0.00-0.16) compared with models using BMID (eFigure 2 in the Supplement). Before vs after HHFKA grade 5 BMID change by free or reduced-price NSLP participation between cohorts was similar (χ^2^_1_ = 1.57, *P* = .21). Associations across unweighted sensitivity models were similar (eTable 7 in the Supplement).

## Discussion

In this cohort study, we estimated associations between the free or reduced-price NSLP and children’s change in BMID before and after the implementation of the HHFKA in 2 cohorts of school-aged children. Before the implementation of the HHFKA, children participating in the free or reduced-price NSLP had a more obesogenic BMI trajectory between grades 1 and 5 than children who did not participate. However, this association was not observed in the recent cohort who entered grade 5 after HHFKA implementation. Associations between free or reduced-price NSLP participation and BMID change between kindergarten to grade 1 (pre-HHFKA implementation period for both cohorts) were similar across cohorts, which provides evidence that implementation of the HHFKA, not cohort differences, explains our results.

Results of research evaluating school meals and child obesity preceding the HHFKA have been mixed, with several reports suggesting that school meals contribute to child obesity,^6,7,8,9,43,47,48^ whereas others do not.^21,49,50^ Among studies with null findings,^49,50^ data limitations^51,52,53^—that is, measurement bias from parent-reported height and weight, small numbers of children, and inability to distinguish body mass growth differences during development—may have biased findings toward the null. Vericker et al^19^ found that the HHFKA reversed the association between the NSLP and child BMI growth for only boys in the ECLS. Yet without quasi-experimental consideration, the authors estimated grade 1 to 3 changes in BMI *z* scores separately for the cohorts, and they did not test whether associations differed across cohorts.^19^

The implementation of the HHFKA increased fruit, vegetable, and whole grain amounts and limited saturated fats. In addition, the HHFKA introduced provisions to improve school environments, such as increasing nutritional requirements of all foods and beverages sold in schools (eg, Smart Snacks^54^). Although school-level provisions may have improved child body mass growth, on average, children in ECLS-K:2011 started school in kindergarten with BMID skewed more toward obesity than ECLS-K:1999 children. Despite the downward trajectory, all ECLS-K:2011 children had a mean BMI in grade 5 that was about as close to the obesity threshold as for fifth graders participating in the free or reduced-price NSLP in ECLS-K:1999. This finding suggests that school policy may need to overcome population-level drivers of child obesity when children may be more predisposed to obesity than children in previous decades.^55^ The gap between participants and nonparticipants may have closed in ECLS-K:2011 because nonparticipants did not experience the BMID decline that their historical counterparts did. This could reflect secular environmental differences (eg, competitive food or beverage availability) in which children in ECLS-K:2011 may have had greater exposure to obesogenic dietary options. Increasing access to school meals with more rigorous nutritional requirements that are culturally pleasing to children may be needed to achieve greater success in reducing child obesity.

Despite arguments against nutrition standards in the HHFKA,^56^ increasing evidence shows that the HHFKA has not reduced school meal participation^57,58^ or students’ acceptance of school lunches.^59^ Moreover, recent studies have found that the HHFKA was associated with improvements in the dietary intake of US school students,^13,16,18^ which is consistent with studies examining nutritional quality after HHFKA implementation.^12,38^ The present study ended in 2016, prior to the 2018 loosening of the nutritional requirements in the HHFKA.^60^ Whether future studies will find support for the HHFKA improving disparities in child body mass growth after 2018, or whether the proposed weakening of the nutrition standards becomes implemented, is unknown.^61^

### Limitations and Strengths

Our study has limitations. To address selection bias of free or reduced-price NSLP participation we estimated BMID change within and between individuals across cohorts. We did not restrict our sample to children who changed free or reduced-price NSLP status, and we used weights to balance cohort differences. However, BMI trajectories may differ as a result of unobserved factors not adjusted for, as acknowledged previously.^21^ Within-cohort and cross-cohort comparisons of associations between NSLP participation and BMID should be interpreted cautiously. We addressed confounding by accounting for latent time-invariant child characteristics, BMID trajectory, and multiple imputation of missing covariate data. The lack of information on full-price NSLP participation after HHFKA implementation in ECLS-K:2011 is a limitation. However, we imputed missing participation information. Although finding statistically significant associations supports our findings, the lack of a cross-cohort difference between BMID change and free or reduced-price NSLP participation does not. However, most fifth graders (94%) in ECLS-K:2011 were categorized as participants, which limited our statistical power to detect a cross-cohort difference. We were unable to assess differential associations by race and ethnicity due to limited samples. In addition, the NSLP may have a longer lagged association with the BMID than we accounted for in our analyses. Our study was also limited by incomplete school breakfast participation information. We did not use survey weights because we selected a sample of students from low-income families; thus, our analytic sample is not nationally representative.

Despite these limitations, direct contrasts in body mass growth before vs after HHFKA implementation enabled us to identify a critical period for HHFKA-associated change in the free or reduce price-NSLP from grades 1 through 5. Our ability to include lagged BMID growth enabled us to estimate BMID change while accounting for children who may have been susceptible to early onset obesity,^62^ either a result of genetic factors or postnatal environments.^63,64^ This ability may have enabled us to detect body mass growth change associated with the NSLP using a measure of BMI that is sensitive to high values. Indeed, the attenuated obesogenic association between the NSLP and BMI *z* score change in grade 5 before HHFKA implementation highlights the limitations in the use of BMI *z* scores. In addition, our findings were robust to clustering by school, adjusting for time-varying mother’s educational level, and adjusting for time-varying school-level percent eligible for the free or reduced-price NSLP.

## Conclusions

In this cohort study using cross-cohort comparisons of children in ECLS-K:1999 and ECLS-K:2011, we observed obesogenic growth of children from kindergarten through grade 5 among participants in the free or reduced-price NSLP before the implementation of the HHFKA that was absent after the implementation, suggesting that the nutritional standards in the HHFKA improved low-income children’s BMI trajectories.

## Notes

Supplement. **eTable 1.** Baseline Characteristics of Analytic Sample Compared to Children Excluded for Missing BMI **eTable 2.** Tested Models and Fit Indices **eTable 3.** Random and Fixed Effects Models by Cohort, Hybrid Model 2b by Cohort, and Hybrid Model 5 by Cohort With Lagged BMID
eMethods.
**eTable 4.** Child, Family, and School Characteristics of ECLS-K:2011 and ECLS-K:1999 Low-Income Child Samples **eTable 5.** Deciles of Numerical Variables of ECLS-K:2011 and ECLS-K:1999 Low-Income Child Samples **eFigure 1.** Path Estimates of Model **eTable 6.** Performance Characteristics of Proposed Full and Simple Imputation Approaches for Predicting Grade 1 Full or Free or Reduced-Price NSLP Participation **eFigure 2.** Path Estimates of Sensitivity Model Using BMI *z* Score **eTable 7.** Free or Reduced-Price NSLP Estimates in Unweighted Sensitivity Models
eReferences
