# Supplementary material for: Association of the Healthy, Hunger-Free Kids Act of 2010 With Body Mass Trajectories of Children in Low-Income Families
Source: JAMA Netw Open. 2022 May 5;5(5):e2210480. doi: 10.1001/jamanetworkopen.2022.10480 (PMC9073566; doi:10.1001/jamanetworkopen.2022.10480)
Supplement: Supplement. — eTable 1. Baseline Characteristics of Analytic Sample Compared to Children Excluded for Missing BMI eTable 2. Tested Models and Fit Indices eTable 3. Random and Fixed Effects Models by Cohort, Hybrid Model 2b by Cohort, and Hybrid Model 5 by Cohort With Lagged BMID eMethods. eTable 4. Child, Family, and School Characteristics of ECLS-K:2011 and ECLS-K:1999 Low-Income Child Samples eTable 5. Deciles of Numerical Variables of ECLS-K:2011 and ECLS-K:1999 Low-Income Child Samples eFigure 1. Path Estimates of Model eTable 6. Performance Characteristics of Proposed Full and Simple Imputation Approaches for Predicting Grade 1 Full or Free or Reduced-Price NSLP Participation eFigure 2. Path Estimates of Sensitivity Model Using BMI z Score eTable 7. Free or Reduced-Price NSLP Estimates in Unweighted Sensitivity Models eReferences [file jamanetwopen-e2210480-s001.pdf]

Supplementary Online Content

Richardson AS, Weden MM, Cabreros I, Datar A. Association of the Healthy, Hunger-Free Kids Act of 2010 with body mass trajectories of children in low-income families. *JAMA Netw Open*. 2022;5(5):e2210480. doi:10.1001/jamanetworkopen.2022.10480

eTable 1. Baseline Characteristics of Analytic Sample Compared to Children Excluded for Missing BMI
eTable 2. Tested Models and Fit Indices
eTable 3. Random and Fixed Effects Models by Cohort, Hybrid Model 2b by Cohort, and Hybrid Model 5 by Cohort With Lagged BMID
eMethods.
eTable 4. Child, Family, and School Characteristics of ECLS-K:2011 and ECLS-K:1999 Low-Income Child Samples
eTable 5. Deciles of Numerical Variables of ECLS-K:2011 and ECLS-K:1999 Low-Income Child Samples
eFigure 1. Path Estimates of Model
eTable 6. Performance Characteristics of Proposed Full and Simple Imputation Approaches for Predicting Grade 1 Full or Free or Reduced-Price NSLP Participation
eFigure 2. Path Estimates of Sensitivity Model Using BMI *z* Score
eTable 7. Free or Reduced-Price NSLP Estimates in Unweighted Sensitivity Models
eReferences.

This supplementary material has been provided by the authors to give readers additional information about their work.

© 2022 Richardson AS et al. *JAMA Network Open*.

eTable 1. Baseline Characteristics of Analytic Sample Compared[1] to Children Excluded for Missing BMI

	ECLS-K:1999			ECLS-K:2011		
**Characteristic**	**Analytic sample (*n*=3,388)**	**Excluded for missing BMI (*n*=583)**	***P*-value for categorical variables<br><br>Difference and 95% confidence interval for continuous variables**	**Analytic sample (*n*=2,570)**	**Excluded for missing BMI (*n*=468)**	***P*-value for categorical variables<br><br>Difference and 95% confidence interval for continuous variables**
Race and ethnicity- No. (%)						
Black non-Hispanic	605 (17.9)	101 (17.3)		280 (10.9)	70 (15.0)	
Hispanic	955 (28.2)	201 (34.5)		1,026 (39.9)	157 (33.6)	
White non-Hispanic	1,418 (41.9)	202 (34.7)		979 (38.1)	180 (38.5)	
Other[2]	409 (12.1)	79 (13.6)	0.003	285 (11.1)	61 (13.0)	0.01
Female- No. (%)	1,696 (50.1)	275 (47.2)		1,222 (47.6)	232 (49.6)	
Male- No. (%)	1,692 (49.9)	308 (52.8)	0.20	1,348 (52.5)	236 (50.4)	0.42
Free or reduced-price National School Lunch Program participation- No. (%)	1,896 (56.0)	308 (52.8)	0.16	1,767 (68.8)	339 (72.4)	0.11
Birthweight in grams	2,017 (448)	1,993 (457)	244 (-160, 648)	3,305 (609)	3,246 (644)	59 (-12, 131)
Mother's educational attainment at kindergarten- No. (%)						
< 9th grade	323 (9.7)	73 (12.7)		278 (10.8)	33 (7.1)	
Grade 9-12	503 (15.1)	87 (15.2)		344 (13.4)	64 (13.7)	
High school or GED	1,333 (40.1)	215 (37.5)		764 (29.7)	144 (30.8)	
Some college	944 (28.4)	152 (26.5)		853 (33.2)	165 (35.3)	
Bachelor's degree	160 (4.8)	41 (7.1)		240 (9.3)	45 (9.6)	
Graduate school	61 (1.8)	6 (1.1)	0.03	91 (3.5)	17 (3.6)	0.29
Household income ($)– mean (SD)	25,159 (17,852)	24,288 (15,846)	871 (-674, 2416)	31,894 (26,568)	30,438 (26,810)	1457 (-1165, 4079)
Family dinners, mean (SD), No./wk	5.8 (1.8)	5.6 (1.9)	0.2 (0.1, 0.3)	5.8 (1.7)	5.9 (1.7)	-0.1 (-0.3, 0.1)
Television, mean (SD), h/d	2.1 (1.4)	2.1 (1.4)	-0.0 (-0.1, 0.1)	2.3 (1.5)	2.2 (1.7)	0.1 (-0.1, 0.2)
School in urban area- No. (%)	1,270 (37.5)	274 (47.0)	<0.001	914 (35.8)	201 (45.4)	<0.001

© 2022 Richardson AS et al. *JAMA Network Open*.

eTable 1. Baseline characteristics of analytic sample compared[1] to children excluded for missing BMI (continued)

	ECLS-K:1999			ECLS-K:2011		
**Characteristic**	**Analytic sample (*n*=3,388)**	**Excluded for missing BMI (*n*=583)**	***P*-value for categorical variables<br><br>Difference and 95% confidence interval for continuous variables**	**Analytic sample (*n*=2,570)**	**Excluded for missing BMI (*n*=468)**	***P*-value for categorical variables<br><br>Difference and 95% confidence interval for continuous variables**
Mother employed at kindergarten- No. (%)	1,258 (42.4)	224 (45.6)	0.63	644 (32.3)	119 (33.2)	0.74

[1] T-tests for continuous variables, Chi-square tests for non-ordinal categorical variables, and Spearman's rank correlation for ordinal categorical variables; Percentages exclude missings

[2] American Indian or Alaska Native, Asian, Native Hawaiian or Pacific Islander, or more than 1 race

© 2022 Richardson AS et al. *JAMA Network Open*.

eTable 2. Tested Models and Fit Indices

Model	Chi-square	Degrees of freedom	CFI	TLI	RMSEA	SABIC
(1) Time-invariant λ	1504.3	38	0.843	0.407	0.114	351747
(2a) Time invariant λ, β*yz*	1757.7	62	0.819	0.58	0.096	351740
(2b) Time invariant β*yz*	141.6	58	0.991	0.978	0.022	349072
(3) Classic random effects, time invariant λ, β*yz* & β*yx*, *COV* (*xit*, ηi) = 0, σε with z	5925.3	126	381	0.292	0.124	89282
(4) Classic fixed effects, time invariant λ, β*yz* & β*yx*, σε with no z	5086.7	335	0.397	-0.002	0.161	313413
(5) Time invariant β*yz* – with BMID lag	19.9	10	0.999	0.99	0.018	280112

λ=factor loading on latent variable eta
B*yz*= coefficient association between outcome and time invariant covariates
B*yx*= coefficient association between outcome and time-varying covariates
σε= error variances
Abbreviations: CFI, comparative fit index, TLI, Tucker Lewis index, RMSEA, root mean square error of approximation, SABIC, sample-size adjusted Bayesian information criteria
The model with the best model fit characteristics for CFI, TLI, and RMSEA before adding lagged BMID association was model 2b. We chose model (5) after adding BMID lags to model (2b) because it has the best model fit for CFI, TLI, and RMSEA. The chosen model does not perform as well with respect to the SABIC as the classic fixed effects model. However, we note that many of the models with smaller SABIC scores have poor fits with respect to all other metrics.

© 2022 Richardson AS et al. *JAMA Network Open*.

eTable 3a. Random effects model by cohort

BMID outcome	ECLS-K:1999 Beta (95% Confidence Interval)	ECLS-K:2011 Beta (95% Confidence Interval)
**Time varying covariates**		
Free or reduced-price NSLP	0.19 (0.04, 0.34)	0.04 (-0.14, 0.22)
Household income ($)	-0.01 (-0.05, 0.03)	-0.06 (-0.09, -0.03)
Family dinners, No./wk	0.01 (-0.03, 0.04)	-0.02 (-0.06, 0.03)
Television, h/d	-0.06 (-0.10, -0.02)	-0.02 (-0.07, 0.03)
School in urban area	-0.03 (-0.35, 0.29)	0.01 (-0.25, 0.25)
Mother employed	0.09 (-0.06, 0.24)	0.05 (-0.12, 0.22)
**Time invariant covariates**		
Female (Male is reference)	-0.80 (-1.07, -0.53)	-0.42 (-0.73, -0.11)
Race or ethnicity (white is reference)		
Black non-Hispanic	0.87 (0.44, 1.29)	0.60 (0.07, 1.12)
Hispanic	0.79 (0.39, 1.20)	0.72 (0.35, 1.09)
Other[1]	0.15 (-0.13, 0.43)	0.15 (-0.13, 0.43)
Birthweight in grams	0.89 (0.62, 1.15)	1.22 (0.88, 1.57)
Mother's educational attainment at kindergarten	-0.07 (-0.20, 0.06)	-0.10 (-0.24, 0.03)

Abbreviations: BMID, BMI difference from 95th percentile, NSLP, National School Lunch Participation

[1] American Indian or Alaska Native, Asian, Native Hawaiian or Pacific Islander, or more than 1 race

eTable 3b. Fixed effects model by cohort

BMID outcome	ECLS-K:1999 Beta (95% Confidence Interval)	ECLS-K:2011 Beta (95% Confidence Interval)
**Time varying covariates**		
Free or reduced-price NSLP	0.14 (-0.03, 0.31)	-0.03 (-0.23, 0.17)
Household income ($)	0.00 (-0.04, 0.04)	-0.05 (-0.09, -0.01)
Family dinners, No./wk	0.02 (-0.01, 0.06)	-0.02 (-0.06, 0.03)
Television, h/d	-0.09 (-0.14, -0.04)	-0.04 (-0.10, 0.02)
School in urban area	-0.12 (-1.06, 0.83)	0.22 (-0.16, 0.60)
Mother employed	-0.01 (-0.17, 0.15)	-0.07 (-0.27, 0.12)
**Latent time invariant variable (ηi) covariance with time varying variables**		
Free or reduced-price NSLP		
Kindergarten	0.10 (0.01, 0.20)	0.06 (-0.03, 0.14)
Grade 1	0.13 (0.05, 0.21)	0.09 (0.03, 0.16)
Grade 5	0.11 (0.04, 0.18)	0.11 (0.05, 0.16)
Household Income ($)		
Kindergarten	-0.48 (-0.98, 0.02)	-0.20 (-0.61, 0.20)
Grade 1	-0.23 (-0.74, 0.29)	-0.30 (-0.64, 0.05)
Grade 5	-0.60 (-0.92, -0.27)	-0.64 (-0.97, -0.31)
Family dinners, No./wk		
Kindergarten	-0.43 (-0.80, -0.07)	-0.11 (-0.44, 0.21)
Grade 1	-0.18 (-0.54, 0.17)	-0.19 (-0.49, 0.11)
Grade 5	-0.20 (-0.52, 0.12)	0.17 (-0.09, 0.43)
Television, h/d		
Kindergarten	0.63 (0.32, 0.93)	0.22 (-0.04, 0.48)
Grade 1	0.25 (0.03, 0.47)	0.25 (-0.03, 0.52)
Grade 5	NA	NA
School in urban area		
Kindergarten	0.08 (-0.15, 0.30)	-0.05 (-0.17, 0.07)
Grade 1	0.10 (-0.14, 0.34)	-0.05 (-0.17, 0.07)
Grade 5	0.08 (-0.14, 0.29)	-0.05 (-0.14, 0.05)
Mother employed		
Kindergarten	0.04 (-0.05, 0.12)	0.12 (0.02, 0.22)
Grade 1	0.14 (0.06, 0.21)	0.09 (-0.00, 0.18)
Grade 5	0.06 (-0.00, 0.13)	0.04 (-0.03, 0.12)

Abbreviations: BMID, BMI difference from 95th percentile, NSLP, National School Lunch Participation, NA, Not applicable

© 2022 Richardson AS et al. *JAMA Network Open*.

eTable 3c. Hybrid model 2b by cohort

BMID outcome	ECLS-K:1999<br>Beta (95% Confidence Interval)	ECLS-K:2011<br>Beta (95% Confidence Interval)
**Time varying covariates**		
Free or reduced-price NSLP		
Kindergarten	0.06 (-0.05, 0.16)	-0.14 (-0.26, -0.02)
Grade 1	-0.04 (-0.16, 0.09)	-0.18 (-0.34, -0.02)
Grade 5	0.62 (0.35, 0.89)	-0.01 (-0.53, 0.52)
Household income ($)		
Kindergarten	-0.01 (-0.03, 0.02)	-0.02 (-0.03, 0.00)
Grade 1	-0.02 (-0.06, 0.01)	-0.02 (-0.05, 0.00)
Grade 5	0.03 (-0.03, 0.09)	-0.05 (-0.10, -0.00)
Family dinners, No./wk		
Kindergarten	0.01 (-0.01, 0.04)	0.01 (-0.02, 0.04)
Grade 1	-0.00 (-0.03, 0.03)	-0.02 (-0.05, 0.01)
Grade 5	0.02 (-0.04, 0.07)	-0.08 (-0.14, -0.01)
Television, h/d		
Kindergarten	-0.01 (-0.04, 0.02)	0.02 (-0.02, 0.05)
Grade 1	-0.00 (-0.04, 0.03)	-0.02 (-0.03, 0.07)
Grade 5	NA	NA
School in urban area		
Kindergarten	-0.08 (-0.58, 0.43)	0.26 (-0.00, 0.52)
Grade 1	-0.07 (-0.72, 0.58)	0.27 (-0.04, 0.58)
Grade 5	-0.20 (-1.09, 0.70)	0.16 (-0.13, 0.44)
Mother employed		
Kindergarten	-0.03 (-0.14, 0.08)	0.01 (-0.10, 0.12)
Grade 1	-0.11 (-0.23, 0.02)	0.07 (-0.05, 0.19)
Grade 5	0.13 (-0.11, 0.36)	0.16 (-0.13, 0.44)
**Time invariant covariates**		
Female (Male is reference)	-0.42 (-0.60, -0.25)	-0.45 (-0.67, -0.24)
Race or ethnicity (white is reference)		
Black non-Hispanic	0.32 (0.05, 0.60)	0.13 (-0.22, 0.48)
Hispanic	0.54 (0.22, 0.85)	0.48 (0.20, 0.76)
Other[1]	0.07 (-0.21, 0.35)	0.04 (-0.30, 0.39)
Birthweight in grams	0.44 (0.24, 0.65)	0.92 (0.61, 1.24)
Mother's educational attainment at kindergarten	0.00 (-0.09, 0.09)	-0.04 (-0.14, 0.06)

© 2022 Richardson AS et al. *JAMA Network Open*.

eTable 3c. Hybrid model 2b by cohort (continued)

BMID outcome	ECLS-K:1999 Beta (95% Confidence Interval)	ECLS-K:2011 Beta (95% Confidence Interval)
**Latent time invariant variable's indicator coefficients λt**		
Kindergarten	1.00	1.00
Grade 1	1.35 (1.31, 1.40)	1.28 (1.24, 1.32)
Grade 5	1.91 (1.83, 1.99)	1.82 (1.74, 1.91)
**Latent time invariant variable (ηi) covariance with time varying variables**		
Free or reduced-price NSLP		
Kindergarten	0.03 (-0.02, 0.07)	0.03 (-0.02, 0.07)
Grade 1	0.05 (0.01, 0.09)	0.05 (0.02, 0.09)
Grade 5	0.02 (-0.03, 0.06)	0.05 (0.02, 0.08)
Household Income ($)		
Kindergarten	-0.20 (-0.50, 0.10)	-0.08 (-0.30, 0.14)
Grade 1	-0.02 (-0.31, 0.28)	-0.15 (-0.35, 0.04)
Grade 5	-0.26 (-0.50, -0.03)	-0.34 (-0.57, -0.10)
Family dinners, No./wk		
Kindergarten	-0.24 (-0.47, -0.01)	-0.06 (-0.26, 0.13)
Grade 1	-0.09 (-0.32, 0.15)	-0.07 (-0.27, 0.13)
Grade 5	-0.08 (-0.30, 0.14)	0.18 (-0.01, 0.37)
Television, h/d		
Kindergarten	0.27 (0.10, 0.45)	0.09 (-0.07, 0.25)
Grade 1	0.06 (-0.07, 0.19)	0.09 (-0.09, 0.27)
Grade 5	NA	NA
School in urban area		
Kindergarten	0.01 (-0.08, 0.11)	-0.06 (-0.13, 0.01)
Grade 1	0.03 (-0.08, 0.13)	-0.06 (-0.13, 0.01)
Grade 5	0.02 (-0.08, 0.11)	-0.05 (-0.12, 0.01)
Mother employed		
Kindergarten	0.04 (-0.01, 0.09)	0.07 (0.01, 0.13)
Grade 1	0.10 (0.05, 0.15)	0.05 (-0.01, 0.10)
Grade 5	0.04 (-0.01, 0.09)	0.03 (-0.03, 0.08)

Abbreviations: BMID, BMI difference from 95th percentile, NSLP, National School Lunch Participation, NA, Not applicable

[1]American Indian or Alaska Native, Asian, Native Hawaiian or Pacific Islander, or more than 1 race

© 2022 Richardson AS et al. *JAMA Network Open*.

eTable 3d. Hybrid model 5 by cohort with lagged BMID

	ECLS-K:1999	ECLS-K:2011
BMID outcome	Beta (95% Confidence Interval)	Beta (95% Confidence Interval)
**Lagged dependent variable coefficients ρt**		
Grade 1 on kindergarten	1.04 (0.74, 1.33)	1.18 (0.89, 1.48)
Grade 5 on grade 1	1.43 (1.33, 1.52)	1.40 (1.32, 1.48)
**Time varying covariates**		
Free or reduced-price NSLP		
Grade 1 on kindergarten	-0.25 (-1.17, 0.68)	0.08 (-1.04, 1.20)
Grade 5 on grade 1	0.54 (0.27, 0.81)	-0.07 (-0.58, 0.45)
Household income ($)		
Grade 1 on kindergarten	0.04 (-0.16, 0.24)	0.09 (-0.15, 0.33)
Grade 5 on grade 1	0.04 (-0.02, 0.10)	-0.04 (-0.09, 0.01)
Family dinners, No./wk		
Grade 1 on kindergarten	0.12 (-0.09, 0.34)	0.10 (-0.20, 0.40)
Grade 5 on grade 1	0.02 (-0.04, 0.08)	-0.07 (-0.14, 0.01)
Television, h/d		
Grade 1 on kindergarten	0.09 (-0.14, 0.31)	0.20 (-0.21, 0.60)
Grade 5 on grade 1	NA	NA
School in urban area		
Grade 1 on kindergarten	0.35 (-0.70, 1.39)	-0.17 (-1.22, 0.87)
Grade 5 on grade 1	-0.28 (-0.60, 0.05)	-0.10 (-0.49, 0.29)
Mother employed		
Grade 1 on kindergarten	0.73 (-0.04, 1.50)	0.18 (-0.68, 1.05)
Grade 5 on grade 1	0.12 (-0.12, 0.35)	0.15 (-0.14, 0.43)
**Time invariant covariates**		
Female (Male is reference)	-0.11 (-0.26, 0.04)	0.14 (-0.00, 0.28)
Race or ethnicity (white is reference)		
Black non-Hispanic	0.16 (-0.08, 0.40)	0.19 (-0.14, 0.52)
Hispanic	0.02 (-0.22, 0.26)	0.05 (-0.20, 0.29)
Other[1]	-0.05 (-0.23, 0.12)	0.05 (-0.20, 0.30)
Birthweight in grams	0.13 (-0.03, 0.29)	-0.05 (-0.32, 0.23)
Mother's educational attainment at kindergarten	-0.08 (-0.15, 0.00)	-0.06 (-0.16, 0.04)
**Latent time invariant variable's indicator coefficients λt**		
Grade 1	1.00	1.00
Grade 5	-0.31 (-0.45, -0.16)	-0.32 (-0.57, -0.08)

© 2022 Richardson AS et al. *JAMA Network Open*.

eTable 3d. Hybrid model 5 by cohort with lagged BMID (continued)

BMID outcome	ECLS-K:1999<br>Beta (95% Confidence Interval)	ECLS-K:2011<br>Beta (95% Confidence Interval)
**Latent time invariant variable (ηi) covariance with time varying variables**		
Free or reduced-price NSLP		
Grade 1	0.06 (-0.09, 0.22)	-0.01 (-0.16, 0.13)
Grade 5	0.03 (-0.03, 0.09)	0.00(-0.05, 0.05)
Household Income ($)		
Grade 1	-0.29 (-0.93, 0.34)	-0.33 (-1.16, 0.49)
Grade 5	-0.18 (-0.41, 0.04)	-0.20 (-0.62, 0.22)
Family dinners, No./wk		
Grade 1	-0.32 (-0.96, 0.32)	-0.32 (-1.16, 0.53)
Grade 5	-0.08 (-0.29, 0.13)	-0.13 (-0.42, 0.17)
Television, h/d		
Grade 1	-0.14 (-0.58, 0.30)	-0.29 (-1.06, 0.48)
Grade 5	NA	NA
School in urban area		
Grade 1	-0.07 (-0.28, 0.14)	0.02 (-0.18, 0.24)
Grade 5	-0.06 (-0.25, 0.13)	0.02 (-0.15, 0.19)
Mother employed		
Grade 1	-0.16 (-0.35, 0.03)	-0.01 (-0.21, 0.19)
Grade 5	-0.06 (-0.14, 0.02)	-0.01 (-0.21, 0.19)

Abbreviations: BMID, BMI difference from 95th percentile, NSLP, National School Lunch Participation, NA, Not applicable

[1] American Indian or Alaska Native, Asian, Native Hawaiian or Pacific Islander, or more than 1 race

© 2022 Richardson AS et al. *JAMA Network Open*.

eMethods.

Structural equation model

We used structural equation modelling (SEM) to evaluate alternative model specifications in which we tested assumptions about covariances between $\boldsymbol{x}_{ijt}$ and η_{ij} and about the time-invariance of coefficients. SEM allows estimation of multiple regressions simultaneously, modeling of time-varying associations and of unmeasured (or latent) variables. SEM estimates a latent variable as a continuous factor using observed variables as indicators of the underlying construct.[1] We tested model fit for a fixed effects model, a random effects model, and a model with time varying and time-invariant coefficients, with and without lagged outcomes. Lagged BMID outcomes are estimated by auto-regression (i.e., current on previous wave).

We identified the preferred model using goodness of model fit along with estimate precision of parameters that distinguished between the model assumptions. A Root Mean Square Error of Approximation (RMSEA) <0.06,[2] Tucker-Lewis Index (TLI)[3] and Comparative Fit Index (CFI) values > .95[2], minimized sample-size adjusted Bayesian information criteria[4] imply the model fits the data well. The modeling commands and illustrative output are provided below. The random and fixed effects models fit poorly (**eTable2**), and their assumptions were invalid as model estimates show in **eTables 3a-3d**. The significant covariance between the latent time-invariant variable η_i and the time-varying covariates in the fixed effects model suggests the random effects model is biased because it does not allow such covariance. Similar to the random effects model, the fixed effects model assumes all coefficients and error variances are equal over time. We find this assumption invalid because we observe different associations between time-varying covariates and BMID in our hybrid model, suggesting the fixed effect model is also biased. Indeed, the fixed effects model failed to identify an association between BMID and free or reduced-price NSLP participation in the pre-HHFKA cohort. The fact that free or reduced-price NSLP doesn't often vary over time within child may have contributed to this failure.

A good fitting model included time-invariant coefficients for time-invariant variables (e.g., gender), and time-varying coefficients for time-varying covariates (**eTable 3c**). The best fitting model improved the model fit with lagged outcomes (**eTable 3d**). We modelled the outcome $BMID_{ijt}$ for each child *i* observed at each age *t (t=0* for kindergarten, *t=1* for grade 1, t=5 at grade 5) in cohort *j* (*j=0* for ECLS-K:1999 or *j=1* for ECLS-K:2011). The covariate of interest $NSLP_{ijt}$ and α_{jt} is the age-by-cohort varying association between NSLP and BMID.

$$BMID_{ijt} = \alpha_{jt}\, NSLP_{ijt} + \rho_{jt} BMID_{ij(t-1)} + \boldsymbol{\beta}_{jt}\boldsymbol{x}_{ijt} + \boldsymbol{\delta}_j \boldsymbol{z}_{ij} + \lambda_{jt}\eta_{ij} + e_{ijt}$$

The model included the lagged effect of $BMID_{ji(t-1)}$ to account for BMID tracking over age. This autoregressive association is denoted with the coefficient ρ_{jt}. The model adjusts for the vector $\boldsymbol{x}_{jit}$ of age-varying covariates (e.g., income) observed at time *t*=1 and *t*=5 and $\boldsymbol{z}_{ji}$ of age-invariant covariates (e.g., birthweight) for each child *i* in their respective cohort *j*. Finally, the model adjusts for a scalar η_{ji} which is defined as the latent, unobserved variables with an age- and cohort-varying coefficient λ_{jt}.

Our three null hypotheses were:

Hypothesis 1: $\alpha_{01} = \alpha_{05} = \alpha_{11} = \alpha_{15} = 0$

Hypothesis 2: $(\alpha_{01} - \alpha_{05}) = 0$ and $(\alpha_{11} - \alpha_{15}) = 0$

Hypothesis 3: $\alpha_{05} = \alpha_{15}$

We acknowledge the "sharp" null hypothesis criticism around a specific value. Accordingly, we prioritized the uncertainty around our parameter estimates that our confidence provided and how well competing models fit the data. Hypothesis 3 assessed whether the association between free or reduced-price NSLP and BMID change at grade 5 differed pre- versus post-HHFKA. We assessed whether the associations were different using Wald's test and because effect modification tests are often under-powered[5], we chose an $\alpha \leq 0.10$.

Entropy balancing and covariate imputation

The purpose of entropy balancing, which is closely related to the widely used propensity score weighting,[6] is both to make the results of downstream analyses less sensitive to model misspecification and reduce the bias of causal estimates.[7,8] This process is related to propensity score weighting[6] and we chose entropy weighting over propensity score balancing because of its ability to balance groups across a large number of variables.

© 2022 Richardson AS et al. *JAMA Network Open*.

In this work, entropy balancing is applied to reweight individuals such that the ECLS-K:1999 and ECLS:K-2011 samples are comparable on child, family and school characteristics. We have balanced both first and second moments of all baseline and time-varying covariates included in the SEM model. Entropy balancing and balance measures are computed using the R package entbal (https://github.com/bvegetabile/entbal). Missing covariate data (reported in **Table 1**) were handled through multiple imputation by chained equations (MICE).[9,10] We produced 10 imputed datasets of entropy-balancing weights, which then served as inputs to weighted SEM models. The core functions described above were executed using the following R commands:

```
# number of imputed datasets
m <- 10

# number of moments to balance
moments <- 2

# multiply impute m complete datasets
imputed <- mice(ecls, m = m, seed = 1)

# set entropy balancing parameters
ebp <- list(exp_type = 'binary', estimand = 'ATE', n_moments = moments, optim_method = 'L-BFGS-B', verbose = T, opt_constraints = c(-250,250), bal_tol = 1e-8, max_iters = 1000)

# obtain entropy balanced weights for each imputed dataset
for(i in 1:m){
ecls_mice <- complete(imputed, i)
weights <- entbal(treatment ~ agemos0 + childrace + female + bwgt + momeduK + inc0 + inc1 + inc5 + famdin0 + famdin1 + famdin5 + tvhrs0 + tvhrs1 + urb0 + urb1 + urb5 + mjob0 + mjob1 + mjob5, data = ecls_mice, eb_pars = ebp)
}
```

Pooled parameter estimates and their variances were then obtained using Rubin's Rules.[11] Supplement eTable 4 summarizes the balance characteristics of the sample before and after entropy balance weights are applied. The computed measures are the mean and standard deviations in each cohort, the standardized difference in means, and the log of the ratio of standard deviations. For binary variables, the standard deviation is defined as $\sqrt{p(1-p)}$, where p is the proportion of individuals with the positive level. We note that, for binary variables, the standard deviation is a deterministic function of the mean. Therefore, balance of the first moment ensures balance of the second moment (and all higher moments). The mother's education variable – which was originally encoded as a six-level ordinal variable – was treated as a continuous variable for the purposes of balancing. This was done to reduce the number of distinct variables requiring balancing. In general, increasing the number of balanced variables increases the design effect incurred due to balancing, which reduces the precision of parameter estimates. Moderate magnitudes of the standardized difference in means or log ratio of standard deviations >0.1[6] indicate disparities in the covariate distributions between ECLS-K:1999 and ECLS-K:2011 cohorts. As can be seen in Panel B, both of these metrics are numerically zero (up to two decimal places) after entropy balancing weights are applied for all measured covariates. These tables represent a single imputed dataset: each of the multiply imputed datasets achieved similar balanced after entropy weights were applied.

SEM modeling commands and illustrative output

In Mplus, we used TYPE=IMPUTATION and weight commands to include the entropy balancing weights. We used the command 'grouping' to estimate the multi-group model by cohort. In the Analysis section of the code, we set iterations to 10,000 and specified 'estimator is mlr'. Convergence criterion was the default value of 0.5 x10^{-4}. To test whether associations differed across cohorts we used the 'Model constraint' and 'Model test' commands. Specifically, we named parameters of interest in the two cohort groups (e.g., BMID at grade 5 on free or reduced-price NSLP at grade 5 (a5) in ECLS-K:1999 and BMID at grade 5 on free or reduced-price NSLP at grade 5 (aa5) in ECLS-K:2011). Then we created new variables of the difference between the two parameters and tested whether the difference variable was equal to zero. Example code and output is below:

© 2022 Richardson AS et al. *JAMA Network Open*.

Model constraint:
new (fifth);

fifth = a5-aa5;

Model test:
fifth=0;

Wald Test of Parameter Constraints

Value	4.122
Degrees of Freedom	1
P-Value	0.0423

To obtain 95% confidence intervals we used the 'cinterval' command in the Output section. Below is an example of the model output (without confidence intervals).

MODEL RESULTS

	Estimate	S.E.	Est./S.E.	Two-Tailed P-Value	Rate of Missing
Group 1999					
ETA BY					
BD951	1.000	0.000	999.000	999.000	0.000
BD955	-0.309	0.074	-4.148	0.000	0.007
BD951 ON					
FRP1	-0.246	0.474	-0.520	0.603	0.004
BD950	1.035	0.150	6.923	0.000	0.015
INC1	0.038	0.101	0.381	0.704	0.014
FAMDIN1	0.124	0.111	1.116	0.264	0.002
TVHRS1	0.086	0.114	0.754	0.451	0.001
URB1	0.347	0.532	0.652	0.514	0.002
MJOB1	0.731	0.391	1.867	0.062	0.012
FEMALE	-0.106	0.076	-1.404	0.160	0.011
BLACK	0.157	0.123	1.281	0.200	0.008
HISP	0.022	0.124	0.174	0.861	0.003
OT	-0.052	0.088	-0.593	0.553	0.003
BWGT	0.127	0.082	1.546	0.122	0.028
MOMEDUK	-0.075	0.039	-1.917	0.055	0.027
BD955 ON					
FRP5	0.536	0.138	3.881	0.000	0.004
BD951	1.425	0.049	28.797	0.000	0.020
INC5	0.042	0.032	1.306	0.192	0.002
FAMDIN5	0.017	0.030	0.568	0.570	0.005
URB5	-0.275	0.167	-1.643	0.100	0.011
MJOB5	0.115	0.120	0.957	0.339	0.017
FEMALE	-0.106	0.076	-1.404	0.160	0.011
BLACK	0.157	0.123	1.281	0.200	0.008
HISP	0.022	0.124	0.174	0.861	0.003
OT	-0.052	0.088	-0.593	0.553	0.003
BWGT	0.127	0.082	1.546	0.122	0.028
MOMEDUK	-0.075	0.039	-1.917	0.055	0.027

eTable 4. Child, Family, and School Characteristics of ECLS-K:2011 and ECLS-K:1999 Low-Income Child Samples

Panel A: Unweighted sample characteristics

Variable	ECLS-K:2011		ECLS-K:1999		Standardized difference in means	Log-Ratio of standard deviations
	Mean	SD	Mean	SD		
Age in months	73.62	4.53	74.64	4.27	-0.23	0.06
Race or ethnicity: Black non-Hispanic	0.11	0.31	0.18	0.38	-0.20	0.10
Race or ethnicity: Hispanic	0.40	0.49	0.28	0.45	0.25	0.24
Race or ethnicity: Other[1]	0.11	0.31	0.12	0.33	-0.03	0.10
Female	0.48	0.50	0.50	0.50	-0.05	0.25
Birthweight	2.02	0.45	2.02	0.45	0.00	0.00
Mother's educational attainment-kindergarten	3.27	1.23	3.10	1.09	0.15	0.13
Logged household income-kindergarten	3.19	2.66	2.52	1.79	0.30	0.40
Logged household income-grade 1	3.19	2.59	2.57	1.56	0.29	0.51
Logged household income-grade 5	3.98	3.10	3.05	2.29	0.34	0.30
Family dinners/week- Kindergarten	5.85	1.73	5.81	1.79	0.02	-0.03
Family dinners, No./wk- grade 1	5.69	1.79	5.86	1.72	-0.09	0.04
Family dinners, No./wk - grade 5	5.58	1.82	5.59	1.80	-0.01	0.01
Television, h/d- kindergarten	2.26	1.52	2.10	1.44	0.10	0.05
Television, h/d- grade 1	1.79	1.36	2.33	1.46	-0.39	-0.07
School in urban area- kindergarten	0.36	0.48	0.37	0.48	-0.04	0.23
School in urban area- grade 1	0.37	0.48	0.37	0.48	-0.02	0.23
School in urban area- grade 5	0.33	0.47	0.37	0.48	-0.09	0.22
Mother employed- kindergarten	0.32	0.47	0.42	0.49	-0.21	0.22
Mother employed- grade 1	0.35	0.48	0.47	0.50	-0.23	0.23
Mother employed- grade 5	0.43	0.50	0.50	0.50	-0.13	0.25

© 2022 Richardson AS et al. *JAMA Network Open*.

eTable 4. eTable 4. Child, Family, and School Characteristics of ECLS-K:2011 and ECLS-K:1999 Low-Income Child Samples (continued)

Panel B: Weighted sample characteristics

	ECLS-K:2011		ECLS-K:1999		Standardized difference in means	Log-Ratio of standard deviations
**Variable**	**Mean**	**SD**	**Mean**	**SD**		
Age in months	74.20	4.42	74.20	4.42	0.00	0.00
Race or ethnicity: Black non-Hispanic	0.15	0.36	0.15	0.36	0.00	0.00
Race or ethnicity: Hispanic	0.33	0.47	0.33	0.47	0.00	0.00
Race or ethnicity: Other[1]	0.12	0.32	0.12	0.32	0.00	0.00
Female	0.49	0.50	0.49	0.50	0.00	0.00
Birthweight	2.02	0.45	2.02	0.45	0.00	0.00
Mother's educational attainment-kindergarten	3.17	1.15	3.17	1.15	0.00	0.00
Logged household income-kindergarten	2.81	2.23	2.81	2.23	0.00	0.00
Logged household income- grade 1	2.84	2.09	2.84	2.09	0.00	0.00
Logged household income- grade 5	3.45	2.71	3.45	2.71	0.00	0.00
Family dinners, No./wk- kindergarten	5.83	1.77	5.83	1.77	0.00	0.00
Family dinners, No./wk - grade 1	5.79	1.75	5.79	1.75	0.00	0.00
Family dinners, No./wk - grade 5	5.59	1.81	5.59	1.81	0.00	0.00
Television, h/d- kindergarten	2.17	1.48	2.17	1.48	0.00	0.00
Television, h/d - grade 1	2.10	1.44	2.10	1.44	0.00	0.00
School in urban area- kindergarten	0.37	0.48	0.37	0.48	0.00	0.00
School in urban area- grade 1	0.37	0.48	0.37	0.48	0.00	0.00
School in urban area- grade 5	0.35	0.48	0.35	0.48	0.00	0.00
Mother employed- kindergarten	0.38	0.49	0.38	0.49	0.00	0.00
Mother employed- grade 1	0.42	0.49	0.42	0.49	0.00	0.00
Mother employed- grade 5	0.47	0.50	0.47	0.50	0.00	0.00

[1] American Indian or Alaska Native, Asian, Native Hawaiian or Pacific Islander, or more than 1 race

© 2022 Richardson AS et al. *JAMA Network Open*.

eTable 5. Deciles of Numerical Variables of ECLS-K:2011 and ECLS-K Low-Income Child Samples

Panel A: Unweighted sample characteristics

		Decile								
**Variable**	**Cohort**	**0.1**	**0.2**	**0.3**	**0.4**	**0.5**	**0.6**	**0.7**	**0.8**	**0.9**
Age in months	1999	69.00	71.00	72.00	73.00	75.00	76.00	77.00	78.00	80.00
Age in months	2011	68.09	69.50	70.68	72.07	73.41	74.73	76.04	77.39	79.04
Birthweight	1999	2.00	2.00	2.00	2.00	2.00	2.00	2.00	2.00	3.00
Birthweight	2011	2.00	2.00	2.00	2.00	2.00	2.00	2.00	2.00	2.00
Mother’s educational attainment-kindergarten	1999	2.00	2.00	3.00	3.00	3.00	3.00	4.00	4.00	4.00
Mother’s educational attainment-kindergarten	2011	1.00	2.00	3.00	3.00	3.00	4.00	4.00	4.00	5.00
Logged household income-kindergarten	1999	0.75	1.25	1.25	1.75	2.25	2.75	2.75	3.75	4.50
Logged household income-kindergarten	2011	0.75	1.25	1.75	2.25	2.75	3.25	3.75	4.50	6.25
Logged household income- grade 1	1999	0.75	1.25	1.75	2.25	2.25	2.75	3.25	3.75	4.50
Logged household income- grade 1	2011	0.75	1.25	1.75	2.25	2.75	3.25	3.75	4.50	6.25
Logged household income- grade 5	1999	0.75	1.25	1.75	2.25	2.75	2.75	3.75	4.50	6.25
Logged household income- grade 5	2011	1.25	1.75	2.25	2.75	3.25	3.75	4.50	6.25	6.25
Family dinners, No./wk - kindergarten	1999	3.00	4.00	5.00	7.00	7.00	7.00	7.00	7.00	7.00
Family dinners, No./wk - kindergarten	2011	3.00	5.00	5.00	7.00	7.00	7.00	7.00	7.00	7.00
Family dinners, No./wk - grade 1	1999	3.00	5.00	5.00	7.00	7.00	7.00	7.00	7.00	7.00
Family dinners, No./wk - grade 1	2011	3.00	4.00	5.00	6.00	7.00	7.00	7.00	7.00	7.00
Family dinners, No./wk - grade 5	1999	3.00	4.00	5.00	5.00	7.00	7.00	7.00	7.00	7.00
Family dinners, No./wk - grade 5	2011	3.00	4.00	5.00	5.00	7.00	7.00	7.00	7.00	7.00
Television, h/d- kindergarten	1999	1.00	1.00	1.00	2.00	2.00	2.00	2.00	3.00	4.00
Television, h/d- kindergarten	2011	0.92	1.00	1.42	1.67	2.00	2.17	3.00	3.00	4.00
Television, h/d- grade 1	1999	1.00	1.00	1.50	2.00	2.00	2.50	3.00	3.00	4.00
Television, h/d- grade 1	2011	0.50	1.00	1.00	1.00	1.50	2.00	2.00	2.50	3.00

© 2022 Richardson AS et al. *JAMA Network Open*.

eTable 5. Deciles of Numerical Variables of ECLS-K:2011 and ECLS-K:1999 Low-Income Child Samples

Panel B: Weighted sample characteristics

		Decile								
**Variable**	**Cohort**	**0.1**	**0.2**	**0.3**	**0.4**	**0.5**	**0.6**	**0.7**	**0.8**	**0.9**
Age in months	1999	69.00	70.00	71.00	73.00	74.00	75.00	77.00	78.00	80.00
Age in months	2011	68.71	70.13	71.54	72.92	74.17	75.42	76.50	77.79	79.23
Birthweight	1999	2.00	2.00	2.00	2.00	2.00	2.00	2.00	2.00	3.00
Birthweight	2011	2.00	2.00	2.00	2.00	2.00	2.00	2.00	2.00	3.00
Mother’s educational attainment-Kindergarten	1999	2.00	2.00	3.00	3.00	3.00	3.00	4.00	4.00	4.00
Mother’s educational attainment-Kindergarten	2011	1.00	2.00	3.00	3.00	3.00	4.00	4.00	4.00	4.00
Logged household income-kindergarten	1999	0.75	1.25	1.75	1.75	2.25	2.75	3.25	3.75	6.25
Logged household income-kindergarten	2011	0.75	1.25	1.75	2.25	2.25	2.75	3.25	3.75	4.50
Logged household income- grade 1	1999	0.75	1.25	1.50	1.75	2.25	2.75	3.25	3.75	6.25
Logged household income- grade 1	2011	0.75	1.25	1.75	2.25	2.25	2.75	3.25	3.75	6.25
Logged household income- grade 5	1999	1.25	1.75	2.25	2.25	2.75	3.25	3.75	4.50	6.25
Logged household income- grade 5	2011	0.75	1.75	1.75	2.25	2.75	3.25	3.75	4.50	6.25
Family dinners, No./wk - kindergarten	1999	3.00	4.00	5.00	7.00	7.00	7.00	7.00	7.00	7.00
Family dinners, No./wk - kindergarten	2011	3.00	5.00	5.00	7.00	7.00	7.00	7.00	7.00	7.00
Family dinners, No./wk - grade 1	1999	3.00	4.00	5.00	6.00	7.00	7.00	7.00	7.00	7.00
Family dinners, No./wk - grade 1	2011	3.00	4.00	5.00	6.00	7.00	7.00	7.00	7.00	7.00
Family dinners, No./wk - grade 5	1999	3.00	4.00	5.00	5.00	7.00	7.00	7.00	7.00	7.00
Family dinners, No./wk - grade 5	2011	3.00	4.00	5.00	5.00	7.00	7.00	7.00	7.00	7.00
Television, h/d- kindergarten	1999	1.00	1.00	1.00	2.00	2.00	2.00	3.00	3.00	4.00
Television, h/d- kindergarten	2011	0.75	1.00	1.25	1.50	2.00	2.00	2.50	3.00	4.00
Television, h/d- grade 1	1999	0.75	1.00	1.25	1.50	2.00	2.00	2.50	3.00	4.00
Television, h/d- grade 1	2011	0.75	1.00	1.00	1.50	2.00	2.00	2.00	3.00	4.00

© 2022 Richardson AS et al. *JAMA Network Open*.

eFigure 1. Path Estimates of Model

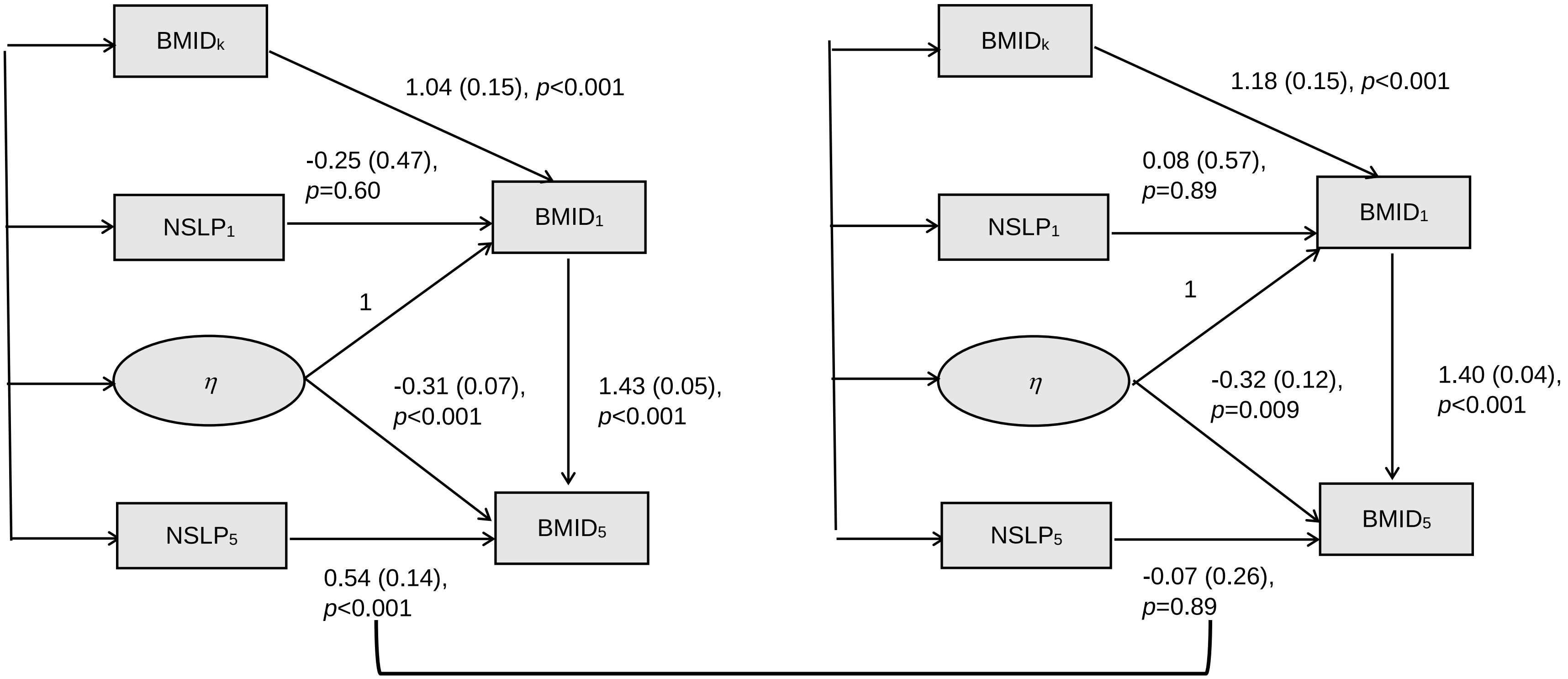

Wald test (e.g., moderation) that association between NSLP and BMID at grade 5 is the same in both cohorts:
$\chi^2 = 4.29$, df=1, $p = 0.04$

Where 0=kindergarten, and t grade 1, and grade 5 waves
Abbreviations: BMID, body mass index distance from obesity (95th percentile), NSLP, Free/reduced-price National School Lunch Program, ECLS, Early Childhood Longitudinal Study, K, Kindergarten.

© 2022 Richardson AS et al. *JAMA Network Open*.

Imputation of ECLS-K:2011 grade 5 full price NSLP

To address unknown full price NSLP participation in ECLS-K:2011, we used imputation to predict grade 5 NSLP participation in ECLS-K:2011 that includes full price. In all years of the ECLS-K cohort and in the kindergarten and grade 1 years of the ECLS-K:2011 cohort, both free or reduced price NSLP participation as well as any NSLP participation (whether full price, free, or reduced) is measured. However, in grade 5 for the ECLS-K:2011 cohort, only free or reduced price NSLP participation is measured. Because grade 5 NSLP participation that included full price lunches was not measured in the ECLS-K:2011 cohort, we imputed grade 5 NSLP participation (whether full price, free, or reduced price) using a model built from the ECLS-K:1999 cohort. We created 100 multiply imputed datasets, and for each we performed the following steps:

1) We fit a logistic regression model on ECLS-K:1999 data to predict a binary indicator of any grade 5 NSLP participation (full price, free, or reduced price) from all model variables observed in both the ECLS-K:1999 and ECLS-K: 2011 cohorts. We note that all grade 5 variables (including grade 5 free or reduced-price NSLP participation) that are observed in both models are included in this predictive model for any NSLP participation.
2) Using this model fit, we then estimate the probability p_i of any grade 5 NSLP participation (full price, free, or reduced price) for each individual in the ECLS-K:2011 cohort.
3) For each individual, any grade 5 NSLP participation (full price, free, or reduced price) is then imputed by drawing a single binary indicator from the distribution $\text{Bernoulli}(p_i)$.

We will refer to the above-described procedure as the *full imputation model* (to be contrasted with two simpler imputation used for comparison purposes below). The purpose of the full imputation model procedure was to leverage data from the ECLS-K:1999 cohort to predict grade 5 full, free/reduced NSLP participation in the ECLS-K:2011 cohort, while appropriately accounting for uncertainty in these estimates by producing multiple imputations of this variable. This procedure is executed using the following commands in R. In this code, the ecls dataset includes data from both cohorts, with the binary variable ec2011 equal to 1 if the individual belongs to the 2011 cohort. Example code is below:

```
# number of imputed datasets
m <- 100

# remove fifth grade lunch (which is missing in 2011) from
# imputation
m0 <- mice(ecls, maxit = 0)
meth <- m0$method
meth[names(meth) %in% c("lunch5")] <- ""
pred <- m0$predictorMatrix
pred[, colnames(pred) %in% c("lunch5")] <- 0

# multiply impute m complete datasets
ecls_mice <- mice(ecls, m = m, predictorMatrix = pred, method = meth)

# initialize matrices for for lunch imputations
lunch5_2011 <-matrix(NA, nrow = dim(filter(ecls, ec2011 == 1))[1], ncol = n_mice)

# predict fifth grade lunch for each imputed dataset
for(i in 1:m){

  # separate data from the 1999 and 2011 cohort
  temp_1999 <- filter(complete(ecls_mice, i), ec2011 == 0)
  temp_2011 <- filter(complete(ecls_mice, i), ec2011 == 1)

  # fit a logistic regression model for fifth grade lunch using 1999 data
  fit <- glm(lunch5 ~ female + bwgt + momeduK + inc0 + inc1 + inc5 + famdin0 + famdin1 + famdin5 + tvhrs0 +
tvhrs1 + urb0 + urb1 + urb5 + mjob0 + mjob1 + mjob5 + ec2011 + lunch0 + lunch1 +_lunch5 + frp0 + frp1 + frp5 +
bd950 + bd951 + bd955 + black + hisp + ot, family = binomial(link = "logit"), data = temp_1999)
```

© 2022 Richardson AS et al. *JAMA Network Open*.

```
  # predict fifth grade lunch using fitted model 2011
  lunch5_preds_2011 <- predict.glm(fit, temp_2011, type = "response",   se.fit = TRUE)$fit
  lunch5_2011[, i] <- rbinom(length(lunch5_preds_2011), 1, lunch5_preds_2011)
}
```

The validity of this procedure relies on the assumption that the predictive model built from the ECLS-K:1999 dataset accurately predicts grade 5 full, free or reduced-price NSLP status in the ECLS:K-2011 dataset. To assess the validity of this hypothesis, we performed the same procedure described above to predict any grade 1 NSLP participation (full, free or reduced-price) in ECLS-K-2011 (which is known) from a model built on ECLS-K:1999 data. We also compared the full imputation approach to two simple imputation approaches. The first procedure imputes grade 1 full, free or reduced-price NSLP participation by setting it equal to the observed kindergarten full, free or reduced-price NSLP status ('kindergarten imputation'). The second simple approach imputes grade 1 full NSLP participation by setting it equal to the observed free or reduced-price NSLP participation in grade 1 (grade 1 free or reduced-price imputation'). Note that free/reduced price-NSLP participation is observed in all grades in the ECLS:K-2011 cohort, while full price NSLP participation is only observed in kindergarten and grade 1.

eTable 6 summarizes the performance of the three imputation approaches for estimation of grade 1 full or free or reduced-price NSLP participation based on (i) overall accuracy (probability of correct imputations), (ii) false positive rate (probability of predicted full or free/ reduced-price NSLP participation given no full or free or reduced-price NSLP participation), and (iii) false negative rate (probability of no predicted full or free or reduced-price NSLP participation given full or free or reduced-price NSLP participation).

The proposed full imputation method has the highest overall accuracy (89.7%), and the lowest false negative rate (2.7%) among the three methods. We note, however, that the false positive rate (65.9%) of the full imputation method is the highest among the three methods. Because the population of NSLP participants ("positives") is much larger than the population of non-NSLP participants ("negatives"), the false-positive/false-negative tradeoff of the imputation method is favorable for overall accuracy. We note that the grade 1 free or reduced-price imputation method has 0% false positive rate. This is because false positives are not possible using this imputation approach: all free or reduced-price participating children are necessarily participating in NSLP. The high overall accuracy of our approach suggests that the assumptions underlying our imputation of grade 5 full or free or reduced-price NSLP status – that the predictive relationships observed in ECLS-K:1999 cohort hold in the ECLS-K:2011 cohort – are not unreasonable.

While predicting full-price NSLP participation separately is another imputation approach, in our view it would be mathematically equivalent to predicting any NSLP participation. Because free or reduced-price lunch in the prediction year implies "any NSLP participation," the modeled coefficient is large, and all individuals receiving free or reduced-price NSLP are imputed as having "any NSLP participation." Our interpretation of the suggested approach is that full-price participation is predicted as a function of the same variables (all model covariates and free or reduced-price participation in all years). Thus, the right hand variables are identical in both models. The outcome the original model (top) is a deterministic function of the suggested outcome (bottom) and a right-hand variable present in both models.

$$\mathrm{logit}(\mathrm{Lunch}_{\mathrm{any}}) = \mathrm{logit}(\mathrm{Lunch}_{\mathrm{full}} + \mathrm{Lunch}_{\mathrm{FRP}}) \sim \beta_X X + \beta_{\mathrm{FRP}}\mathrm{Lunch}_{\mathrm{FRP}}$$
$$\mathrm{logit}(\mathrm{Lunch}_{\mathrm{full}}) \sim \beta'_X X + \beta'_{\mathrm{FRP}}\mathrm{Lunch}_{\mathrm{FRP}}$$

where FRP=free or reduced-price NLSP

Throughout our analyses, we use the full imputation model to impute the missing grade 5 NSLP participation (full or free or reduced-price).

eTable 6. Performance Characteristics of Proposed Full and Simple Imputation Approaches for Estimating Grade 1 Full or Free or Reduced-Price NSLP Participation

Performance metric	Full imputation	Kindergarten imputation	Grade 1free or reduced-price imputation
Overall accuracy	89.7%	84.0%	88.3%
False Positive Rate	65.9%	31.6%	0.0%
False Negative Rate	2.7%	13.9%	13.3%
Proportion predicted grade 1 lunch participation compared to true grade 1 lunch participation of 2,257/2,570 (87.8%)	93.5%	79.5%	76.2%

© 2022 Richardson AS et al. *JAMA Network Open*.

eFigure 2. Path Estimates of Sensitivity Model Using BMI *z* score

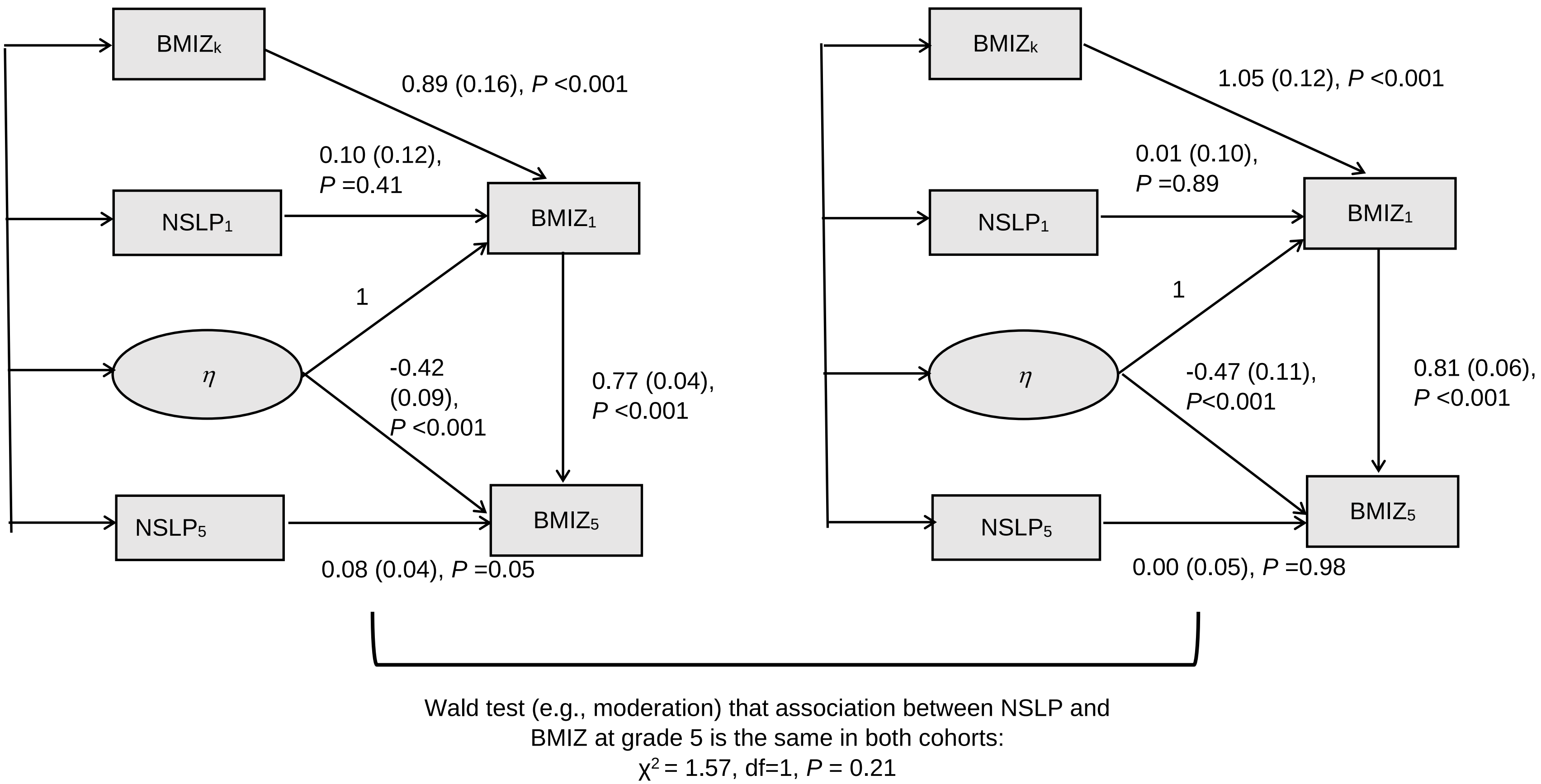

Where 0=kindergarten, and t grade 1, and grade 5 waves
Abbreviations: BMIZ, body mass index *z* score, NSLP, Free/reduced-price National School Lunch Program, ECLS, Early Childhood Longitudinal Study, K, Kindergarten.

© 2022 Richardson AS et al. *JAMA Network Open*.

Additional Sensitivity Analyses

We also estimated sensitivity models with BMI *z* scores. In a set of unweighted sensitivity models we estimated:

1) our main model;

2) clustering on baseline school given the study sampling design;

3) adjusting for time-varying mother's education since some mothers may complete degrees while their children are in elementary school; and

4) adjusting for time varying school-level % students eligible for free or reduced-price lunch which was reported by school administrators (0-24%, 25-49%, 50-74%, and 75-100%) to address potential changes between the two cohorts that could have changed the balance of unobserved factors across NSLP participants and non-participants, such as the Community Eligibility Provision (implemented nationally in school year 2014–15). Model estimates of free or reduced-price NSLP and BMID changes are in eTable 7.

© 2022 Richardson AS et al. *JAMA Network Open*.

eTable7. Free or Reduced-Price NSLP Estimates in Unweighted Sensitivity Models

	ECLS-K:1999	ECLS-K:2011
**BMID outcome**	**Beta (95% Confidence Interval)**	**Beta (95% Confidence Interval)**
**Model 1: main model**		
Free or reduced-price NSLP		
Grade 1	-0.15 (-0.98, 0.68)	0.01 (-0.76, 0.77)
Grade 5	0.40 (0.16, 0.65)	0.14 (-0.22, 0.50)
**Model 2: main model clustered on baseline school**		
Free or reduced-price NSLP		
Grade 1	-0.15 (-1.02, 0.71)	0.03 (-0.77, 0.83)
Grade 5	0.40 (0.15, 0.66)	0.11 (-0.30, 0.51)
**Model 3: main model adjusted for time-varying mother's education**		
Free or reduced-price NSLP		
Grade 1	-0.08 (-0.92, 0.76)	-0.10 (-0.96, 0.76)
Grade 5	0.39 (0.13, 0.64)	0.16 (-0.19, 0.52)
**Model 4: main model adjusted for time-varying school-level % eligible for free or reduced-price NSLP**		
Free or reduced-price NSLP		
Grade 1	-0.03 (-0.88, 0.82)	-0.02 (-0.86, 0.82)
Grade 5	0.38 (0.13, 0.63)	0.06 (-0.31, 0.43)

Abbreviations: BMID, BMI difference from 95th percentile, NSLP, National School Lunch Participation

© 2022 Richardson AS et al. *JAMA Network Open*.

eReferences.

1. Bollen KA. Latent variables in psychology and the social sciences. *Annu Rev Psychol.* 2002;53:605-634.
2. Hu L, Bentler PM. Cutoff criteria for fit indexes in covariance structure analysis: conventional criteria versus new alternatives. *Struct Equ Modeling.* 1999;6(1):1-55.
3. Tucker LR, Lewis C. Reliability Coefficient for Maximum Likelihood Factor-Analysis. *Psychometrika.* 1973;38(1):1-10.
4. Haughton DMA, Oud JHL, Jansen RARG. Information and other criteria in structural equation model selection. *Commun Stat-Simul C.* 1997;26(4):1477-1516.
5. Aiken LS WS. *Multiple Regression: Testing and Interpreting Interactions.* Newbury Park, CA: SAGE Publications, Inc.; 1991.
6. Austin PC. An Introduction to Propensity Score Methods for Reducing the Effects of Confounding in Observational Studies. *Multivar Behav Res.* 2011;46(3):399-424.
7. Hainmueller J. Entropy Balancing for Causal Effects: A Multivariate Reweighting Method to Produce Balanced Samples in Observational Studies. *Polit Anal.* 2012;20(1):25-46.
8. Ho DE, Imai K, King G, et al. Matching as nonparametric preprocessing for reducing model dependence in parametric causal inference. *Polit Anal.* 2007;15(3):199-236.
9. Coffman DL, Zhou JX, Cai XZ. Comparison of methods for handling covariate missingness in propensity score estimation with a binary exposure. *Bmc Medical Research Methodology.* 2020;20(1).
10. van Buuren S, Groothuis-Oudshoorn K. mice: Multivariate Imputation by Chained Equations in R. *J Stat Softw.* 2011;45(3):1-67.
11. Rubin DB, Schenker N. Multiple Imputation for Interval Estimation from Simple Random Samples with Ignorable Nonresponse. *Journal of the American Statistical Association.* 1986;81(394):366-374.

© 2022 Richardson AS et al. *JAMA Network Open*.
